# Loss of ADAM15 prevents necroptosis induction by partial RIPK1 degradation due to enhanced TNF-R1 surface expression and basal caspase-8 activation

**DOI:** 10.1186/s12964-025-02530-3

**Published:** 2025-12-04

**Authors:** S. Braun, K. Knackfuß, T. Ziesmann, L. Mlinzk, A. Goerg, J. Frankenheim, A. Walter, W. Schneider-Brachert, U. Distler, J. Fritsch

**Affiliations:** 1 Department of Infection Prevention and Infectious Diseases, University Hospital of Regensburg, Regensburg, Germany; 2 Institute for Immunology, University Medical Center of the Johannes-Gutenberg University, Mainz, Germany; 3 Institute for Molecular Medicine, University of Lübeck, Lübeck, Germany

**Keywords:** ADAM15, Death receptor signaling, Cell death, Necroptosis, Apoptosis, Proteomics

## Abstract

**Background:**

Cell death and survival processes must be tightly regulated to ensure proper tissue homeostasis and prevent excessive inflammation and tissue damage. Death receptors, including TNF-R1, can induce either immunogenic (necroptosis) or non-immunogenic (apoptosis) cell death and relay proliferative / cell survival signaling by activating NFκB and MAPK cascades. In a recent report, we identified the metalloproteinase ADAM15 as a possible TNF-responding enzyme, leading to the hypothesis that it regulates either cell survival or death cascades.

**Methods:**

CRISPR/Cas-9 was used to knock out the *adam15* gene. Loss of gene expression was validated by Western blot and flow cytometry in U937 and Jurkat cells. NFκB, MAPK signaling, and cell death cascades were monitored by Western blot, flow cytometry, and enzyme assays. A bottom-up proteome analysis was performed to elucidate cellular processes affected by ADAM15 loss. The subcellular localization of ADAM15 was monitored by microscopy and immuno-magnetic fractionation.

**Results:**

We identified ADAM15 as a regulator of necroptosis, leaving apoptosis and cell survival signaling unaffected. Loss of ADAM15 resulted in abrogated necroptosis, as evidenced by the application of death ligands TNF, TRAIL, FasL, and TL1a, as well as the BH3 mimetic Obatoclax. We observed enhanced basal Caspase-8 activity, which was not cytotoxic, and partial RIPK1 proteolysis. The loss of ADAM15 was verified in a proteome screen, which revealed alterations in various molecular pathways, including autophagy, organelle trafficking, and sorting. We observed ADAM15 in intracellular compartments, which in part have a lysosomal protein signature. We observed enhanced surface expression of TNF-R1, proposing it as a possible ADAM15 substrate.

**Conclusions:**

ADAM15 is a previously unknown regulator of necroptosis, likely due to its role in modulating intracellular organelle sorting processes. Its proteolytic activity and possible scaffolding capacity for recruiting adaptor molecules make it a veritable drug target. The activation or deactivation of ADAM15 may be exploited to modulate various disease conditions.

**Supplementary Information:**

The online version contains supplementary material available at 10.1186/s12964-025-02530-3.

## Abstract (plain language summary)

What we discovered about cell death control

Human bodies constantly balance cell survival and death to stay healthy. One key player is TNF-R1—a "death receptor" that responds to inflammation signals. When activated, it initiates a chain reaction that can either keep cells alive or trigger their destruction.

We focused on a protein called ADAM15, discovered in a previous screening. Usually, cells use two main death pathways:

• Apoptosis: An orderly, programmed cell dismantling

• Necroptosis: A more explosive, inflammatory death

Key findings:

1. When we removed ADAM15 from human cells:

○ Necroptosis vanished (even when triggered by multiple signals)

○ Apoptosis and survival pathways still worked normally

2. ADAM15 affects critical death regulators:

○ ADAM15 regulates the presence of TNF-R1 on the cell surface

○ RIPK1 (a death pathway "switch") gets partially cut by another protein called caspase-8

○ This cutting blocks necroptosis but leaves other functions intact

3. ADAM15 also influences:

○ Cellular"shipping systems" (organelle movement)

○ Protein levels of other death regulators

Why it matters:

ADAM15 acts as a specialized traffic controller for cell death. In its absence, cells lose their ability to undergo inflammatory necroptosis while maintaining other functions. This reveals a new layer of control in life-or-death cellular decisions, with potential implications for understanding the pathogenesis of inflammatory diseases.

**Supplementary Information:**

The online version contains supplementary material available at 10.1186/s12964-025-02530-3.

## Background

Tumor necrosis factor receptor 1 (TNF-R1) is a key mediator of extrinsic cell death and survival, driving pro-inflammatory host responses. It is the eponymous member of a family of receptor-ligand pairs that can trigger cell survival and different forms of cell death, hence called death receptors [19]. The binding of tumor necrosis factor (TNF-alpha) to its receptor TNF-R1 mediates the formation of receptor-bound *complex I* in lipid rafts in the cellular plasma membrane. Activation of NFκB signaling is pro-inflammatory and must thus be tightly regulated. *Complex I* formation is controlled by differential post-translational modification of many of the involved proteins (e.g., TNF-R1, RIPK1, cIAPs) by phosphorylation, ubiquitination, and palmitoylation [5, 19, 24]. In the case of inhibited NFκB signaling or inhibited synthesis of survival proteins, the balance shifts in favor of cell death induction. The protein composition of *complex I* is remodeled to form *complex IIa/b*. *Complex IIa* can be either receptor-bound (containing TRADD, FADD, and caspase-8) when TNF-R1 is endocytosed and forms ‘TNF-receptosomes’, which mature towards lysosomes for apoptosis amplification via lysosomal and mitochondrial outer membrane permeabilization (MOMP). Alternatively, *complex II* dissociates from the receptor, presumably involving proteolytic processing of the receptor by ADAM17 and γ-secretase [5]. *Complex IIb* comprises phosphorylated RIPK1, RIPK3, FADD, and caspase-8. Active caspase-8 acts as an initiator caspase, promoting apoptosis induction. Its activity ensures a block of necroptosis (*aka* ‘regulated necrosis’) by degradation of otherwise available RIPK1 and RIPK3. In the absence of caspase-8 activity, complex IIc, also known as the necrosome, is formed. RIPK1 phosphorylates RIPK3, which in turn results in MLKL phosphorylation and oligomerization, ultimately leading to cytolysis due to plasma membrane pore formation. The latter process can be reversed by endocytosis of MLKL-pore-containing membrane areas [5, 27]. RIPK1 and MLKL must both undergo S-acylation to unleash their necroptotic potential [58, 59, 89]. In pyroptotic death, which also relies on the formation of membrane pores, the S-acylation of the effector protein gasdermin D was recently reported [48].

We showed that S-acylation of TNF-R1 is a prerequisite for its plasma membrane translocation, and de-acylation, involving APT2 activity, is required for lipid raft translocation and NFκB pathway induction. In this study, we performed a proteome screen to identify possible S-acylated proteins, revealing alterations in the palmitoylation state upon the addition of TNF [62].

Among the identified proteins was the enzyme a disintegrin and metalloproteinase 15 (ADAM15), which had not been previously associated with TNF-mediated signal transduction. ADAM proteinases are a family of membrane-tethered metalloproteinases involved in releasing extracellular proteins (i.e., cytokines, growth factors, receptors, and adhesion molecules) via ectodomain shedding. ADAM proteinases have been reported to regulate various biological processes, and functions related to intracellular organelles have also been proposed [39]. Knockdown of ADAM15 revealed functional TNF-induced NFκB and MAPK signaling as well as apoptosis induction. Notably, necroptosis could not be induced by various stimuli known to activate this pathway. Protein loss was validated using proteomics, which revealed altered abundances of additional death-regulating proteins that could be confirmed by Western blot. In parallel, we observed an altered band pattern for caspase-8, indicating a low level of constitutive but not cytotoxic activity, which we verified by activity assays. Similarly, we observed altered band patterns for RIPK1. We assumed that RIPK1 might be partially cleaved by caspase-8, resulting in blocked necroptosis but still functional NFκB, MAPK, and apoptosis. A proteome screen revealed differential regulation of proteins in various biological processes, including organelle formation, trafficking, fusion, and the death receptor TNF-R1. Taken together, we identified ADAM15 as a novel regulator of necroptotic cell death, which warrants further investigation in the future.

## Methods

### Cell culture

U937 and Jurkat cells were cultivated using RPMI (Gibco) supplemented with 10% FBS (Gibco) and 5% Sodium-Pyruvate (Gibco) without antibiotics at 37 °C, 5% CO_2_. Cell lines were purchased from DMSZ and routinely screened for identity and mycoplasma infection.

### Protein knockdown

For CRISPR-mediated knockdown, we used commercial Cas9 (IDT) loaded with crRNA:tracrRNA duplexes as recommended by the manufacturer’s protocol (Alt-R CRISPR-Cas9, IDT). Two ADAM15 gene targeting crRNAs (Hs.Cas9.ADAM15.1.AA: ACTGACCACCCGAGTGCCAT; Hs.Cas9.ADAM15.1.AB: TAATTGGGAGATCGTCCTGA) were used to enhance knockout performance, as recommended [67]. Complexes were delivered to the nuclei of 5 × 10^3^ cells using the nucleofection kits (U937: Kit C; Jurkat: Kit V) following the manufacturer’s instructions (Amaxa). Upon nucleofection, pools of cells were screened by WB and flow cytometry for reduced ADAM15 levels and then used for the analyses. No single-cell clones were used to avoid artifacts resulting from clonal expansion from a single cell.

### Cell death analysis – Annexin V/7AAD staining

The assay was performed by seeding 1 × 10^6^ cells/mL. After incubating the cells with the death-inducing agents for the indicated time points, cells were stained following the Muse® Annexin V & Dead Cell Kit’s instructions (Cytek). Samples were measured using the Guava MUSE device (Luminex). 2,000–5,000 cells were measured per condition. Alternatively, 250 µl of carefully resuspended cells were sedimented, washed with PBS (5 min, 500 × g, 4 °C), and stained for 20 min in the dark after adding 22 µl staining mix (20 µl binding buffer, 1 µl FITC-Annexin V, 1 µl 7-AAD; BioLegend). 100 µl binding buffer was added, and samples were measured using a Guava easyCyte (Cytek) and guavaSoft (4.5.25, Cytek) for data analysis. Statistical analyses were performed using GraphPad Prism (v10): Data were normally distributed; a two-way ANOVA with Tukey's multiple comparisons test was used.

Unless indicated otherwise, cell death-inducing compounds were used at the following concentrations: 100 ng/mL TNF (provided by D. Männel, Regensburg), 50 ng/mL KillerTRAIL (ALX-201–073–3020, Enzo Life Sciences), 100 ng/mL TL1a (1319-TL-010, bio-techne), or 75 ng/mL FasL [71]. Cycloheximide (CHX: 2.5 µM; API-03, VWR) and zVAD-fmk (50 µM; S7023, Selleckchem) were added 30 min before the ligand. Obatoclax (GX15-070: 2.5 µM; S6709, Selleckchem). Necrostatin-1 (5 µM; #2324, Tocris/bio-techne).

### Western blot

1 × 10^6^ cells/mL cells were treated with the respective death-inducing compounds for the indicated times to monitor cell death induction. Unstimulated and stimulated cells were sedimented and washed with PBS (5 min, 500 × g). Cell pellets were lysed in 30 µl modified RIPA buffer (50 mM TRIS–HCl [pH 7.5], 150 mM NaCl, 1% NP-40, 1% Triton X-100, 1 mM EDTA, 0.25% Na-deoxycholate), containing a protease inhibitor cocktail (Sigma-Aldrich), followed by 20 s sonication at 4 °C and 5 min sedimentation at 1500 × g. The supernatant was used for BCA protein quantification (Pierce, #23,225). Samples were prepared immediately before SDS-PAGE. For SDS-PAGE, 12.5% PAA gels were used. To detect MLKL-oligomers, 10% PAA gels were used, and DTT was omitted from the sample buffer. Proteins were blotted onto a PVDF membrane (Carl-Roth). The membranes were blocked with 5% skimmed milk in TBST and incubated overnight with the primary antibodies, diluted 1:1,000 in 5% skimmed milk. The peroxidase-conjugated secondary antibodies were incubated for 1 h, diluted 1:10,000 in 5% skimmed milk. Blots were imaged using ECL (Sigma-Aldrich) and the ImageQuant800 device (Cytiva). Where shown, band densities were quantified using ImageJ (version 1.54p) and displayed as protein of interest to loading control (e.g., actin) ratios.

### Primary antibodies

ADAM15 (MAB935, R&D Systems), ADAM15 (HPA011633, Sigma-Aldrich). Cell Signaling: IκB (#4814S), pMAPK (#4370S), PARP-1 (#9542S), cleaved Caspase-3 (#9661S), full-length caspase-3 (#9668S), MLKL (#14993S), RIPK1 (#3493S), RIPK3 (#10188S), MMP9 (#13,667), xIAP (#2045S), TNF-R1 (#3736S), DR4 (#42533S), DR5 (#8074S), Cathepsin D (#69,854), CoxIV (#69,854), ERp72 (#5033S), Optn (#58981S), RNF31/HOIP (#99633S), Diap (#14634S), Tryptase (#92215S), GPNMB (#38313S). Santa Cruz Biotechnology: CD95 (sc-715), cIAP (sc-7943), HSP70 (sc-27), HSP90 (sc-69703), Acid ceramidase (sc-518165), Elf1 (sc-13396), Nucleoporin p62 (sc-166870), HOIL-1/RBCK1 (sc-365523). Lamp-1 (9835–01, Southern biotech), Lamp-2 (9840–01, Southern biotech). RIPK1 (610,459, BD Biosciences). Caspase-8 (ALX- 804–242-C100, Enzo life Sciences). ThermoFisher). Proteintech: Actin-HRP (HRP-60008), Tubulin-HRP (HRP-66031), GAPDH-HRP (HRP-60004). *Secondary antibodies*: anti-mouse-HRP (715–035–150, Jackson ImmunoResearch), anti-rabbit-HRP (111–035–144, Jackson ImmunoResearch).

### Flow cytometry

Since ADAM15 can be located both inside cells and on the cell surface [46], we utilized the inside stain kit and protocol (130–090–477, Miltenyi). 1 × 10^6^ cells were sedimented, washed with PBS (10 min, 300 × g), and resuspended in 250 µl of inside fix. After 20 min of incubation in the dark, cells were washed (5 min, 300 × g) and permeabilized by adding 1 mL of the inside perm, followed by sedimentation (5 min, 300 × g). The cell pellet was resuspended in 98 µl of perm solution, then 2 µl of primary antibody (MAB935) was added, mixed, and incubated for 10 min at RT in the dark. After washing with 1 mL inside perm (5 min, 300 × g), secondary anti-rabbit-AlexaFluor488 (A21206, ThermoFisher) diluted 1:100 in inside perm was added for another 10 min incubation at RT in the dark. After washing, the cells were resuspended in 150 µl PBS, and samples were measured using a Guava easyCyte (Cytek) and guavaSoft (4.5.25, Cytek) for data analysis.

For the quantification of DR and ADAM15 surface expression, the following biotinylated antibodies were used, diluted 1:200 in PBS: CD120a/TNF-R1 (130–106–358, Miltenyi Biotec), CD261/DR4 (103–109-084, Miltenyi Biotec), CD262/DR5 (103–097–303, Miltenyi Biotec), CD95 (103–113-067, Miltenyi Biotec), DR3 (130–105-075, Miltenyi Biotec), ADAM15 (BAF935, R&D Systems). Labeling was performed by staining 5 × 10^5^ Cells in 100 µl PBS for 10 min on ice, followed by 2 × washing and Streptavidin AlexaFluor 488 (S11223, Thermo Fisher) was added (2 mg/mL Stock diluted 1:100 in PBS) for 10 min on ice and 2 × washing. Finally, the cells were resuspended in 150 µl PBS, and samples were measured (5000 cells per condition) using a Guava easyCyte (Cytek) and guavaSoft (4.5.25, Cytek) for data analysis.

### Caspase-8 activity assay

To quantify caspase-8 activity, 5 × 10^5^ cells were incubated with 1 µl FITC-IETD-fmk (88–7005, ThermoFisher) in 300 µl PBS for 30 min under cell culture conditions. Cells were sedimented (5 min, 500 × g), washed twice in 500 µl washing buffer, and resuspended in 150 µl PBS. Fluorescence intensity was quantified using a Guava easyCyte (Cytek) and guavaSoft (4.5.25, Cytek) for data analysis.

### Immunofluorescence

The assay was performed using 1 × 10^6^ cells, which were sedimented (750 × g, 5 min, 4 °C) and then washed using cold PBS. Cells were fixed in 80 µl 3.5% PFA/PBS for 20 min on ice, followed by washing twice in cold PBS. Cells were then permeabilized in 0.2% Saponin/0.1% BSA/PBS for 20 min on ice, followed by washing twice in cold PBS.

The primary antibodies (ADAM15: MAB935, R&D Systems; Lamp-1: #L1418S, Sigma Aldrich) were diluted 1:100 in 100 µL of cold 0.1% BSA/PBS and incubated for 60 min on ice, followed by two washes in cold PBS. The secondary dye-conjugated antibodies (anti-rabbit AF594: 2,563,675, Invitrogen) and anti-mouse AF488: 2,533,878, Invitrogen) were added diluted 1:200 in 100 mL cold 0.1% BSA/PBS and incubated for 60 min in the dark, followed by two washing steps in cold PBS. Cells were then dropped on object slides, allowed to sit and sediment for 30 min, before the supernatant was carefully aspirated. Before completely dried, mounting medium (including DAPI) and a cover slide were added and allowed to harden overnight at 4 °C in the dark. Images were acquired using a BZ-X810 fluorescence microscope (Keyence). To quantify colocalization of ADAM15 and Lamp-1, the Coloc 2 plugin of ImageJ (version 1.54p) was used. The Manders’ tM1/tM2 values (threshold: Costes method) are provided for the respective images.

### Organelle isolation

Lysosomes were isolated following our established protocol [25]. In brief, 2 × 10^8^ cells were sedimented, washed in PBS, and homogenized using a cell homogenizer (Isobiotec, Heidelberg, Germany) with 8 micron or 10 micron spheres and 20 × strokes in a 1 ml volume of homogenization buffer. Homogenates were pre-cleared by centrifugation for 5 min at 1,500 × g. Then the supernatant was loaded on an OptiPrep (Sigma-Aldrich) cushion and centrifuged for 60 min at 100,000 × g. The derived organelle-containing layer (750 µl) was incubated with 5 µl anti-Lamp-1 (#L1418S, Sigma Aldrich) and then subjected to magnetic separation as detailed before [25]. Protein content of the magnetically purified material was quantified using BCA, followed by WB analysis. 4 µg of protein was loaded per lane.

### Proteomics sample preparation

1 × 10^6^ untreated U937 wt and ΔADAM15 cells were washed twice with PBS (5 min, 750 × g) and cell pellets frozen at −80°C until further processing. Cells were subsequently lysed in 400 µL of an urea-based buffer (7 M urea, 2 M thiourea, 5 mM dithiothreitol (DTT), 2% (w/v) CHAPS). Lysis was further promoted by sonication at 4 °C for 15 min using a Bioruptor (Diagenode, Liège, Belgium). The protein concentration was determined using the Pierce 660 nm protein assay (Thermo Fisher Scientific) according to the manufacturer´s protocol. Proteins (corresponding to approx. 20 µg) were digested using a modified filter-aided sample preparation (FASP) protocol as detailed before [5, 80]. In brief, samples were transferred onto spin filter columns (Nanosep centrifugal devices with Omega membrane, 30 kDa MWCO; Pall, Port Washington, NY). Afterwards, detergents were removed by washing the samples (membrane) three times with a buffer containing 8 M urea. After reduction and alkylation by DTT and iodoacetamide (IAA), excess IAA was quenched with DTT, and the membrane was washed three times with 50 mM NH_4_HCO_3_. Afterwards, proteins were digested overnight at 37 °C with trypsin (Trypsin Gold, Promega, Madison, WI) using an enzyme-to-protein ratio of 1:50 (w/w). After digestion, peptides were recovered by centrifugation and two additional washes with 50 mM NH_4_HCO_3_. Combined flow-throughs were acidified with trifluoroacetic acid (TFA) to a final concentration of 1% (v/v) TFA and lyophilized. Purified peptides were reconstituted in 0.1% (v/v) formic acid (FA) for LC–MS analysis.

### Liquid-chromatography mass spectrometry (LC–MS)

LC–MS analyses were performed on an Ultimate 3000 RSLCnano LC system (Thermo Fisher Scientific) coupled to an Orbitrap Exploris 480 instrument platform (Thermo Fisher Scientific). Tryptic peptides were first loaded onto a PEPMAP100 C18 5 µm 0.3 × 5 mm trap column (Thermo Fisher Scientific) and subsequently separated on an HSS-T3 C18 1.8 μm, 75 μm × 250 mm analytical reversed-phase column (Waters Corporation). Mobile phase A was water containing 0.1% (v/v) formic acid and 3% (v/v) DMSO. Peptides were separated running a gradient of 2–35% mobile phase B (0.1% (v/v) formic acid, 3% (v/v) DMSO in ACN) over 40 min at a flow rate of 300 nL/min. Total analysis time was 60 min including wash and column re-equilibration steps. Column temperature was set to 55 °C. The following settings were used for mass spectrometric analysis of eluting peptides on the Orbitrap Exploris 480 instrument platform: Spray voltage was set to 1.9 kV, the funnel RF level to 40, and heated capillary temperature was at 275 °C. Data were acquired in DIA mode. Full MS resolution was set to 120,000 at *m/z* 200 and full MS automated gain control (AGC) target to 300% with a maximum injection time (IT) of 20 ms. Mass range was set to *m/z* 345–1250. Fragment ion spectra were acquired with an AGC target value of 1000%. In total, 21 windows with varying sizes (adjusted to precursor density) were used with an overlap of 0.5 Th. Resolution was set to 30,000 and IT was determined automatically (“auto mode”). Normalized collision energy was fixed at 27%. All data were acquired in profile mode using positive polarity.

### Raw data processing

MS raw data were processed using DIA-NN (version 1.9.2) [5] applying the default parameters for library-free database search. Data were searched using a custom compiled database containing the UniProtKB entries of the human reference proteome and a list of common contaminants (version release June 2024, 20,436 entries). For peptide identification and in-silico library generation, trypsin was set as protease allowing one missed cleavage. Carbamidomethylation was set as fixed modification and the maximum number of variable modifications was set to zero. The peptide length ranged between 7–30 amino acids. The precursor *m/z* range was set to 300–1,800, and the product ion *m/*z range to 200–1,800. As quantification strategy we applied the “QuantUMS (high accuracy)” mode with RT-dependent median-based cross-run normalization enabled. We used the build-in algorithm of DIA-NN to automatically optimize MS2 and MS1 mass accuracies and scan window size. Peptide precursor FDRs were controlled below 1%.

### Statistical and downstream data analysis

In the final proteome datasets, proteins had to be identified by at least two peptides. Statistical analysis of the data was conducted using Student’s t-test, which was corrected by the Benjamini-Hochberg (BH) method for multiple hypothesis testing (FDR of 0.01). Additionally, differentially expressed proteins had to display a log2(FC) of 0.3 or −0.3. The t-test was conducted using stats.ttest_ind from the Python (version 3.10.11) [76] module statsmodels (version 0.14.0) [66]. The volcano plot was created using the packages readxl (version 1.4.5) [79], tidyverse (version 2.0.0) [78], and ggrepel (version 0.9.6) [68] in R (version 4.5.0)(R Core Team (2025). R: A Language and Environment for Statistical Computing. R Foundation for Statistical Computing, Vienna, Austria. < https://www.R-project.org/ >.). The enrichment network plots were created in R (version 4.5.0) using the packages topGO (version 2.59.0) [61], readxl (version 1.4.5), clusterProfiler (version 4.16.0)[84], and enrichplot (version 1.28.0) [87]. The GO enrichment was conducted using enrichGO and the biological processes gene ontology of humans (version 3.21.0) (Carlson M (2025). org.Hs.eg.db: Genome wide annotation for Human.) using 0.01 as cutoffs for both p-value and q-value and Benjamini-Hochberg (BH) correction for multiple hypothesis testing. As input the p-value sof the Student’s t-test and the log2(FC) were used to specify the proteins of interest. Enrichment network plots of the top terms were created using the cnetplot function.

### Data availability

The mass spectrometry proteomics data have been deposited to the ProteomeXchange Consortium (http://proteomecentral.proteomexchange.org) via the jPOST partner repository [56] with the dataset identifiers PXD066056 (ProteomeXchange) and JPST003922 (jPOST). To review the data: Go to: [https://repository.jpostdb.org/preview/300089052687116bd50a22](https://repository.jpostdb.org/preview/300089052687116bd50a22) Access key: 4853 All source code and data files are available from the authors upon request.

### Exogenous expression of RIPK1

pcDNA3-FLAG-RIPK1 was a gift from J. Song (Addgene #78,842). For the expression of mutant (L320A/D323N) and c-terminal flag-tagged RIPK1, the construct was synthesized and cloned into pcDNA3.1_Zeo (GeneArt). 5 × 10^4^ U937 ADAM15 cells were transfected using Amaxa nucleofection Kit C (Lonza), following the manufacturer's protocol. One day post-transfection, cells were incubated in the presence of Zeocin (R25001, Thermo Fisher Scientific). The expression of RIPK was analyzed by Western blot, and cell death was quantified. Viability was quantified using annexin V/7AAD staining.

## Results

### Knockdown of ADAM15 does not alter TNF-induced survival pathways

**Fig. 1:**
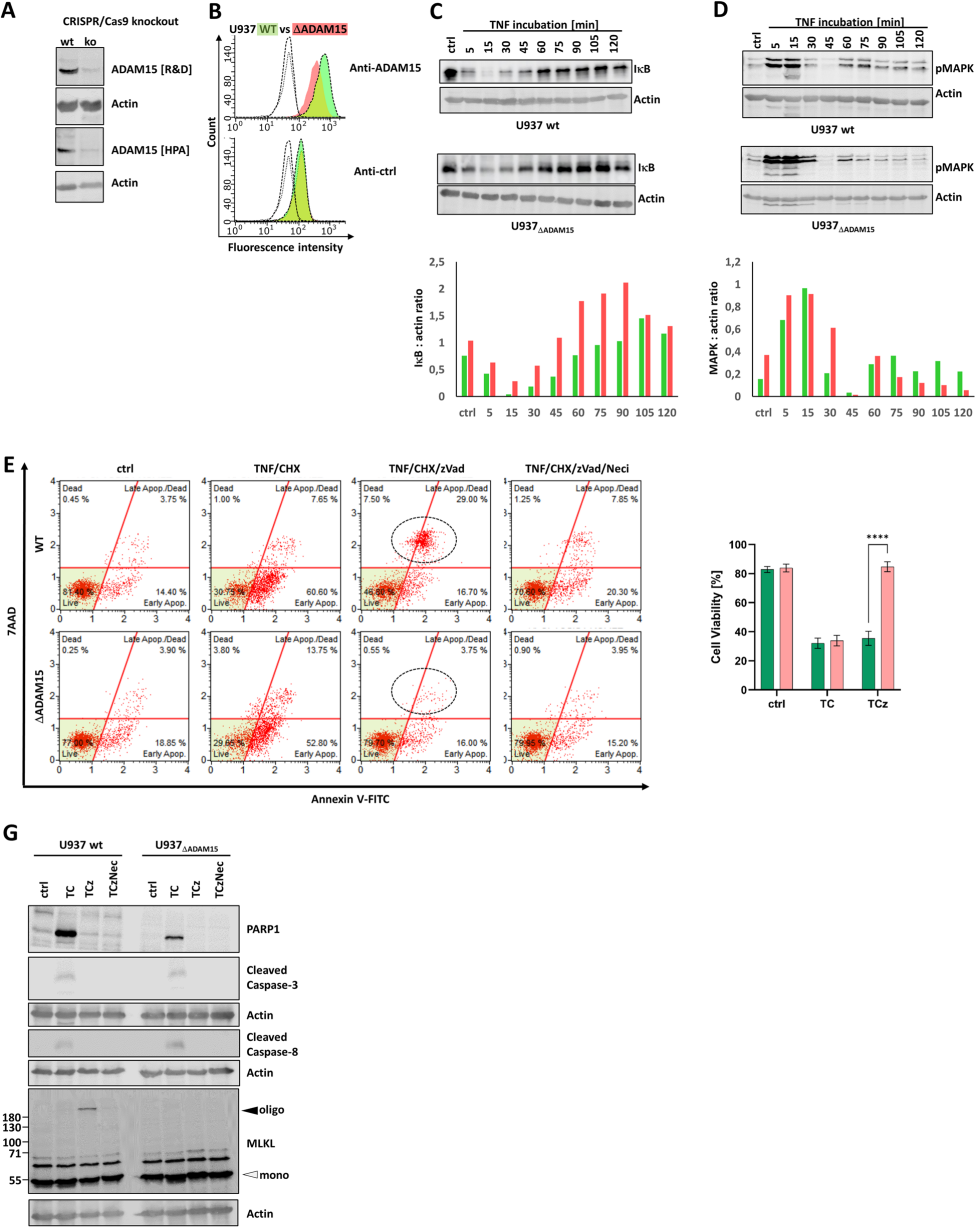
ADAM15 knockdown affects necroptosis but not apoptosis, NFκB, and MAPK signaling in U937 cells. **a** CRISPR Cas-9 mediated knockout of ADAM15 was monitored by WB using two different antibodies (R&D: MAB935 and HPA: HPA011633). Knockout (ko) cells are further referred to as ΔADAM15. Actin served as a loading control. *n* = 3 experiments performed. **b** ADAM15 knockout was validated by flow cytometry. The green curve represents staining using an anti-ADAM15 antibody (upper panel) or an isotype control (lower panel) in U937 wt cells. The red curve represents staining in U937_ΔADAM15_ cells. The dashed non-coloured curves represent unstained cells. Right-shifted curves indicate higher fluorescence intensity. Representative data of *n* = 3 experiments are shown. **c** The degradation of IκB was monitored by WB. Band densities were quantified, and IkB:actin ratios are shown (green: U937 wt; red: U937 ΔADAM15). Representing *n* = 3 independent experiments. **d** The activation/phosphorylation of MAPK was monitored by WB. Band densities were quantified, and MAPK:actin ratios are shown (green: U937 wt; red: U937 ΔADAM15). Representative data of n = 3 experiments are shown. **e** Representative scatter plots of flow cytometry-based cell death analyses. The x-axis shows changes in fluorescence intensity due to increased Annexin V-FITC binding. The y-axis indicates increased fluorescence of 7AAD due to binding to DNA. Multiple (*n* = 9) assays were quantified (right panel; green bars: WT; red bars: ΔADAM15). *: *p* = 0,05; ** *p* = 0,01; *** *p* = 0,001. **f** WB was used to monitor TC-mediated apoptosis (PARP-1 and caspases-3 and −8) and TCz-mediated necroptosis (MLKL oligomer). n = 3 experiments performed. Actin serves as a loading control

The CRISPR/Cas-9-mediated ADAM15 knockdown resulted in a cell pool for which protein expression was monitored and compared to that of U937wt cells by Western blot (Fig. 1A). The absence of ADAM15 was validated by flow cytometry (Fig. 1B); knockout cells for these experiments are referred to as U937_ΔADAM15_. Both U937 wt and U937_ΔADAM15_ cells showed the same growth behavior. However, we observed that after longer passaging (> 5 months), the overall vitality of U937_ΔADAM15_ decreased moderately (not shown).

As we identified ADAM15 as a TNF-responsive protein due to its palmitoylation state [62], we next aimed to investigate the possible roles of the protein in TNF signaling. TNF can activate different cell death and survival pathways. The latter are mediated by activation of the NFκB and MAPK cascades. To investigate the potential impact on TNF-mediated NFκB and MAPK activation, cells were incubated for up to 120 min with TNF. Degradation of IκB as a surrogate for NFκB activation and phosphorylation of MAPK(p44/42) was monitored by Western blot (Fig. 1C** +** D), revealing no significant changes in activation kinetics. The signal for IκB remained moderately intense in U937_ΔADAM15_ cells compared to the wild type, suggesting a slightly dampened effect in NFκB signaling.

#### Knockdown of ADAM15 does not alter TNF-induced apoptosis but mitigates the induction of necroptosis

Next, we investigated the induction of extrinsic apoptosis (TNF/CHX: TC) and necroptosis (TNF/CHX/zVAD: TCz). TNF is the activator, while the addition of CHX is required to sensitize cells for apoptosis by blocking the synthesis of anti-apoptotic factors. When the pan-caspase inhibitor zVAD is added, RIPK1 cannot be degraded by caspase-8, shifting cell death towards necroptosis. Cell death induction was quantified in U937 wt and U937_ΔADAM15_ cells by flow cytometry, adding an Annexin V-FITC conjugate to monitor its binding to surface-exposed phosphatidyl serine (PS), as a marker of (early) apoptosis induction. The usually non-membrane-permeant DNA-binding dye 7AAD was added to monitor plasma membrane leakiness, which occurs either during late apoptosis or necroptosis (MLKL-pores). Figure 1E shows representative scatter plots of multiple analyses. Necrostatin-1 (Neci) was used as a necroptosis inhibitor. Figure 1F shows statistical analysis of multiple assays.

Next, we monitored activation of marker proteins for apoptosis (cleaved Caspases-3, −8, and PARP-1) and necroptosis (MLKL-oligomerization) by Western blot (Fig. 1G). In both U937 wt and ΔADAM15 cells, caspases and PARP-1 are activated under apoptotic (TC) but not necroptotic (TCz) conditions. In U937 wt cells, the molecular weight shift due to MLKL-oligomerization occurs, but is absent in U937_ΔADAM15_ cells.

Taken together, we could show that the absence of ADAM15 mitigates TNF-induced necroptosis.

#### Knockdown of ADAM15 affects TRAIL, TL1a, FasL, and Obatoclax-mediated necroptosis in U937 cells.

**Fig. 2:**
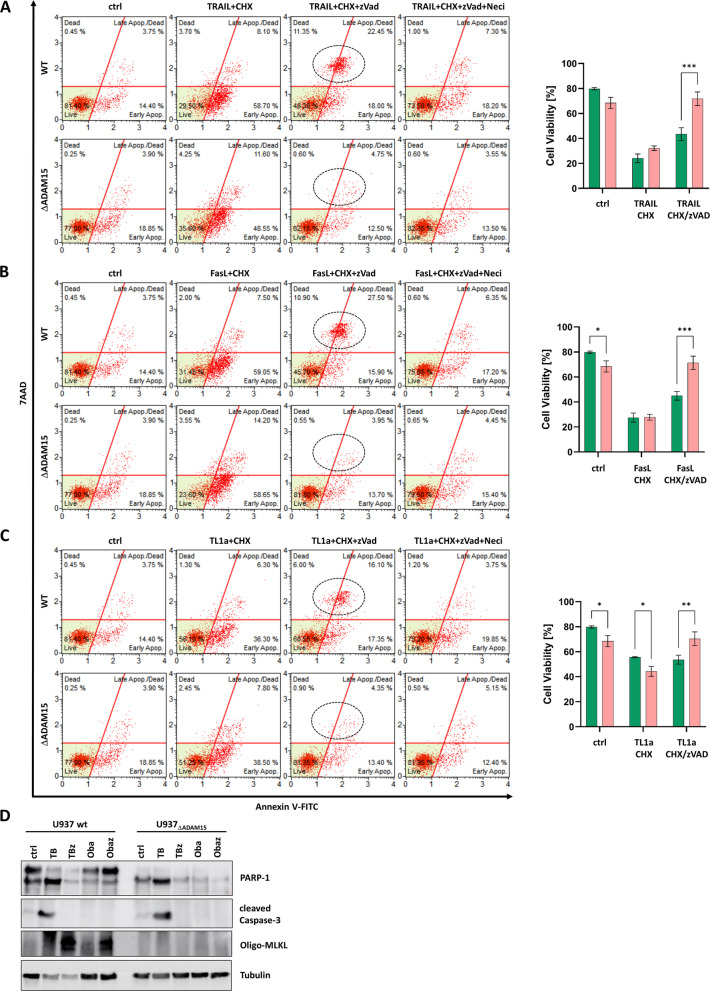
ADAM15 knockdown affects necroptosis but not apoptosis induction in U937 cells mediated by TRAIL, FasL, TL1a, and Obatoclax. **a-c** Representative scatter plots of flow cytometry-based cell death analyses upon incubation with TRAIL (a), FasL (b), or TL1a (**c**) The x-axis shows changes in fluorescence intensity due to increased Annexin V-FITC binding. The y-axis indicates increased fluorescence of 7AAD due to binding to DNA. Multiple (*n* = 3 per ligand) assays were quantified (right panels; green bars: WT; red bars: ΔADAM15) **d** Obatoclax mediated apoptosis (Oba) and necroptosis (Obaz) compared to TNF/BV-6 (TB: apoptosis) and TNF/BV-6/zVAD (TBz: necroptosis) were monitored by WB in U937 wt (left panel) and U937_ΔADAM15_ cells (right panel). Represents *n* = 3 independent experiments. Tubulin serves as a loading control

While TNF and TNF-R1 are the name-giving members of a family of receptor-ligand pairs, not all of these can induce cell death. This capacity requires the presence of an intracellular death domain (DD). This is the case in the TNF related apoptosis inducing ligand (TRAIL) receptors TRAIL-R1 (death receptor 4; DR4; CD261) and TRAIL-R2 (death receptor 5; DR5; CD262), Tumor necrosis factor-like ligand 1 A (TL1a) receptor (death receptor 3; DR3; CD263), and Fas-ligand-receptor (CD95, apo-1-receptor), hence called death receptors (DR). All DR can mediate both apoptosis and necroptosis upon ligand binding, which we monitored by flow cytometry in U937 wild-type and ADAM15-deficient cells (Fig. 2A-C). Statistical analyses of multiple assays are shown on the right side. In all cases, we observed apoptotic responses, whereas necroptosis induction was absent.

Cell death can also be triggered receptor-independently. The experimental anticancer drug Obatoclax (GX15-070) is a Bcl-2 antagonist that has been reported to trigger both apoptotic and necroptotic cell death [3]. We tested this in U937 cells using Western blot. As also tested in preclinical studies, we used the IAP inhibitor BV-6 instead of the protein synthesis inhibitor CHX to sensitize cells for TNF-mediated death in this series of experiments. Analyzing combinations, e.g., TNF with BV-6 and zVAD, showed no difference compared to CHX treatment (not shown). Both U937wt and ΔADAM15 cells showed cleavage of Caspase-3 and PARP-1, and thus apoptosis induction, in response to treatment with TNF and BV-6 but not with Obatoclax. Treatment with TNF/BV-6/zVAD induced MLKL oligomerization in U937 wt but not in ΔADAM15 cells.

The application of Obatoclax alone did not induce apoptosis in any of the cell lines. At the same time, co-treatment of Obatoclax and zVAD resulted in MLKL oligomerization in U937 wt but not in ΔADAM15 cells. (Fig. 2D). Due to its high auto-fluorescence, flow cytometry cannot monitor Obatoclax-treated cells [5].

Together, the loss of ADAM15 not only prevents necroptosis induced by TNF but also by other DR and Obatoclax.

#### Knockdown of ADAM15 alters the expression profiles of multiple proteins

**Fig. 3:**
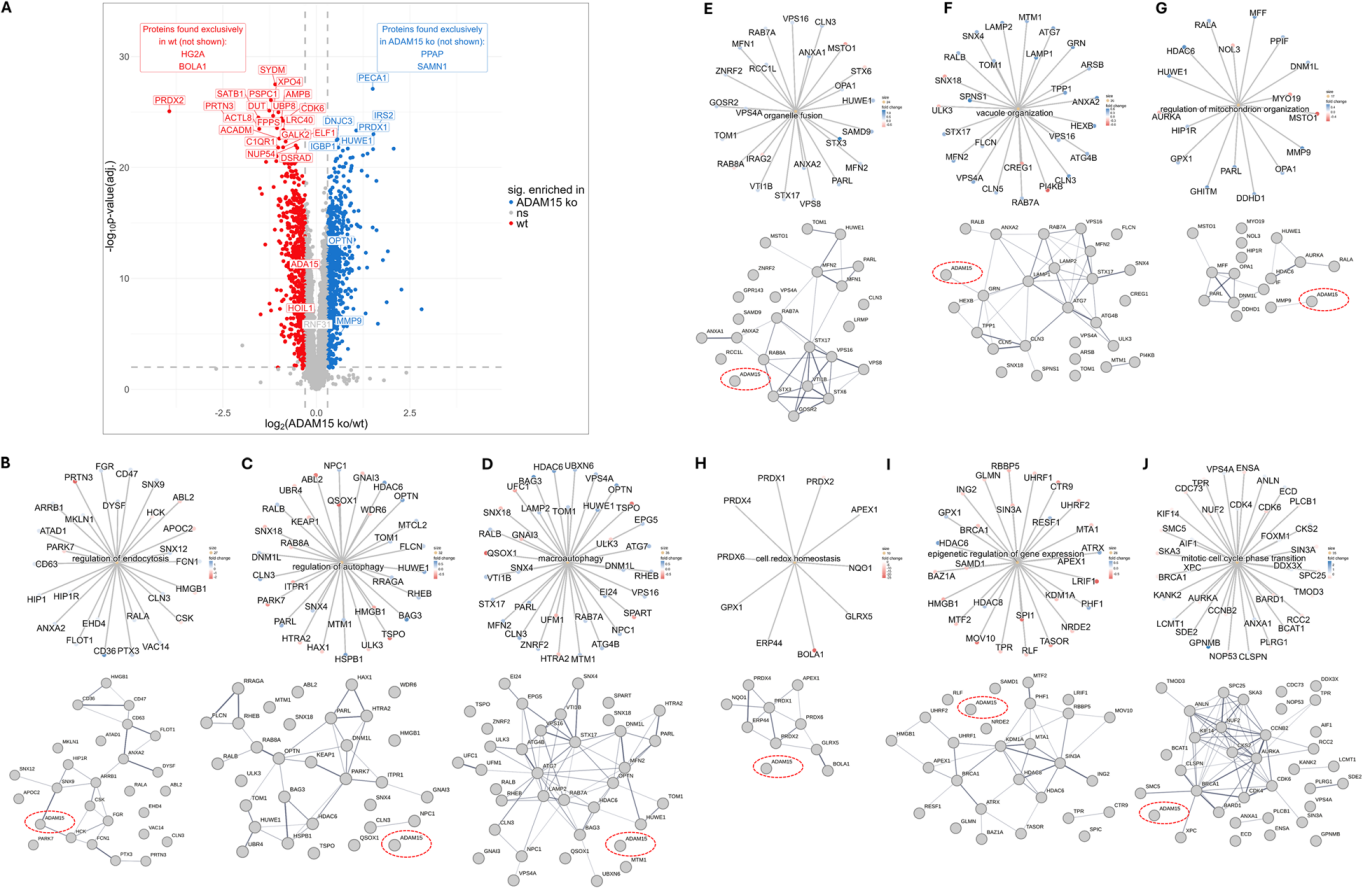
Shotgun proteome analysis reveals regulated proteins upon ADAM15 knockout in U937 wt and U937_ΔADAM15_ cells.** a** Volcano plot (p-value: 0.01; fdr: 0.01; fold-change cutoff 1: 0.3; fold-change cutoff 2: −0.3; min. samples for significance 0.75) shows proteins enriched in wt cells (red) versus U937_ΔADAM15_ cells. The top 25 modulated proteins are indicated along with the selected proteins. The top annotated GSEA groups are shown: **b** ‘Regulation of endocytosis’, **c** ‘Regulation of autophagy’, **d** ‘Macroautophagy’, **e** ‘Organelle fusion’, **f** ‘Vacuole organization’, **g** ‘Regulation of mitochondrion organization’, **h** ‘Cell redox homeostasis’, **i** ‘Epigenetic regulation of gene expression’, and **j** ‘Mitotic cell cycle phase transition’. The upper panel shows the respective GO-termini, encircled by the involved proteins (Fold change is color-coded). The lower panels show the respective proteins as STRING-DB (V12.0) based networks. ADAM15 is highlighted for each network (dashed red circle)

To pinpoint how the loss of ADAM15 results in abrogated necroptosis, we performed a bottom-up proteomics analysis comparing basal protein levels in U937 wt versus U937_ΔADAM15_ cells. Figure 3A displays differentially regulated proteins between the two conditions, highlighting the top 25 regulated proteins (red: enriched in WT, blue: enriched in ΔADAM15). In addition to ADAM15, MMP9, which is known to be upregulated in ADAM15-lacking cells [18], and Optineurin (OPTN), which is a frequently described necroptosis-regulating protein [31], are highlighted. HOIL-1, which regulates TNF-signaling and is part of the linear ubiquitin chain assembly complex (LUBAC), was enriched in wt cells. Its interaction partner RNF31 was, however, not regulated.

GSEA analysis revealed several interconnected molecular pathways affected by ADAM15 knockdown. Among the twelve most altered pathways were the regulation of endocytosis (Fig. 3B), regulation of macro-/autophagy (Fig. 3C** + **D), organelle/vesicle/membrane fusion (Fig. 3E), vacuole organization (Fig. 3F), the regulation of mitochondrion organization (Fig. 3G), cell redox homeostasis (Fig. 3H), epigenetic/negative regulation of gene expression (Fig. 3I), and mitotic cell cycle phase transition (Fig. 3J). Proteins assigned to the respective GO terms, including information on their regulation, are shown in the respective upper panels. The protein networks formed are shown in the respective lower panels, based on the STRING database [73]. ADAM15 is included in the networks (dashed red circle) to highlight known or predicted interactions, which is the case for ADAM15-HCK and ADAM15-SNX9 (Fig. 3B), ADAM15-GRN (Fig. 3F), and ADAM15-MMP9 (Fig. 3G).

**Fig. 4:**
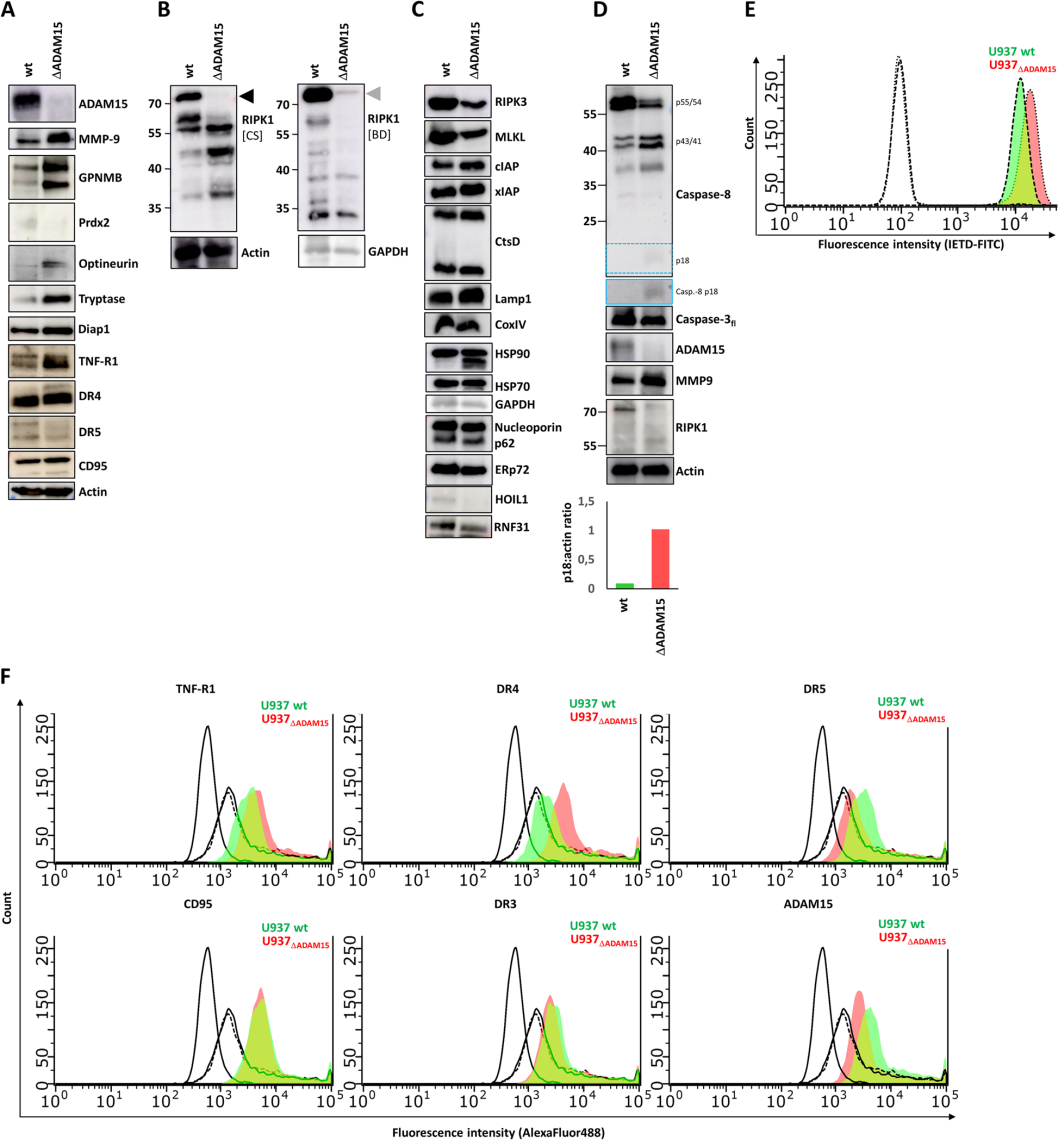
Analysis of differential protein expression by WB and caspase-8 activity. **a** Depicts the validation of up- and down-modulation of selected proteins from the proteome analysis. **b** Representative (of *n* = 3) WB for RIPK1 probed with two different antibodies (cell signaling: #3493S and BD: 610,459). **c** Representative (of *n* = 3) WB analysis of various cell death-associated and non-related proteins.** d** Caspase-8 appears partially active in U937_ΔADAM15_ compared to wt cells, indicated by reduced p55/44 and increased p43/41 and p18 (blue box – enhanced contrast). **e** Validation of increased basal Caspase-8 activity by flow cytometry. Dashed colorless curves indicate no staining. The green curve indicates wt cells stained with IETD-FITC, and the red curve indicates U937 ADAM15 cells with IETD-FITC. X-axis shift to the right side indicates increased fluorescence due to binding of IETD to active caspase-8 (p-18). Representative of *n* = 3 experiments. **f** Flow cytometry-based analysis of death receptor and ADAM15 surface expression. The non-coloured curves represent unstained and Strep-AF488-stained cells, the green curve WT, and the red curve ADAM15 cells. Representative data of *n* = 3 experiments

Based on the proteome data, the modulation of selected proteins, i.e., those most up- or downregulated, including ADAM15 knockout and concomitant MMP9 upregulation, was verified by Western blot and is shown in Fig. 4A-D. We additionally performed WB to detect protein levels of death receptors: TNF-R1 was enriched in ADAM15 cells according to the proteome data, while DR4, DR5, and CD95 were not altered. In line, TNF-R1 showed elevated levels, while the others were not changed. DR3 was neither detected by MS nor by WB.

Interestingly, we observed altered band patterns for RIPK1, revealing reduced levels of full-length protein and the appearance of lower molecular weight bands. No alterations in protein abundance were quantified by MS. RIPK3 and MLKL showed a moderate reduction in U937_ΔADAM15_ cells by Western blot analysis; however, no changes were detected by MS. HSP90, another protein showing an altered band pattern in U937_ΔADAM15_ cells, was previously described to regulate necroptosis [32, 41]. In an earlier study, we observed the appearance of an additional HSP90β band upon TNF treatment, which was due to partial pro-apoptotic protein cleavage [22, 29].

We observed small amounts of cleaved caspase-8, suggesting a partially active enzyme (Fig. 4D), which is insufficient to initiate cell death immediately. However, this may explain our observation that the vitality of U937_ΔADAM15_ decreased over time. Moderately enhanced caspase-8 activity in U937_ΔADAM15_ cells could be verified using an enzyme activity assay (Fig. 4E). As we recently reported, detoxification of low amounts of active caspase-8 is mediated via a non-canonical autophagy process [30], which may be dysregulated due to ADAM15 loss.

One reason for low caspase-8 activation without ligand binding could be the upregulation of death receptors, which may result in enhanced clustering and thus also caspase activation [5]. Indeed, the proteome data and WB revealed a higher abundance of TNF-R1 protein in U937_ΔADAM15_ cells compared to the wild type. Flow cytometry also showed enhanced TNF-R1 surface expression (Fig. 4F). Interestingly, DR4 displayed enhanced surface expression, while DR5 was moderately reduced on the cell surface of ADAM15-lacking cells. CD95 and DR3 were not altered.

Together, we verified the changes in abundance of various proteins, including TNF-R1, which could be a direct substrate of ADAM15.

#### ADAM15 knockdown in Jurkat cells also abrogates necroptotic signaling

**Fig. 5:**
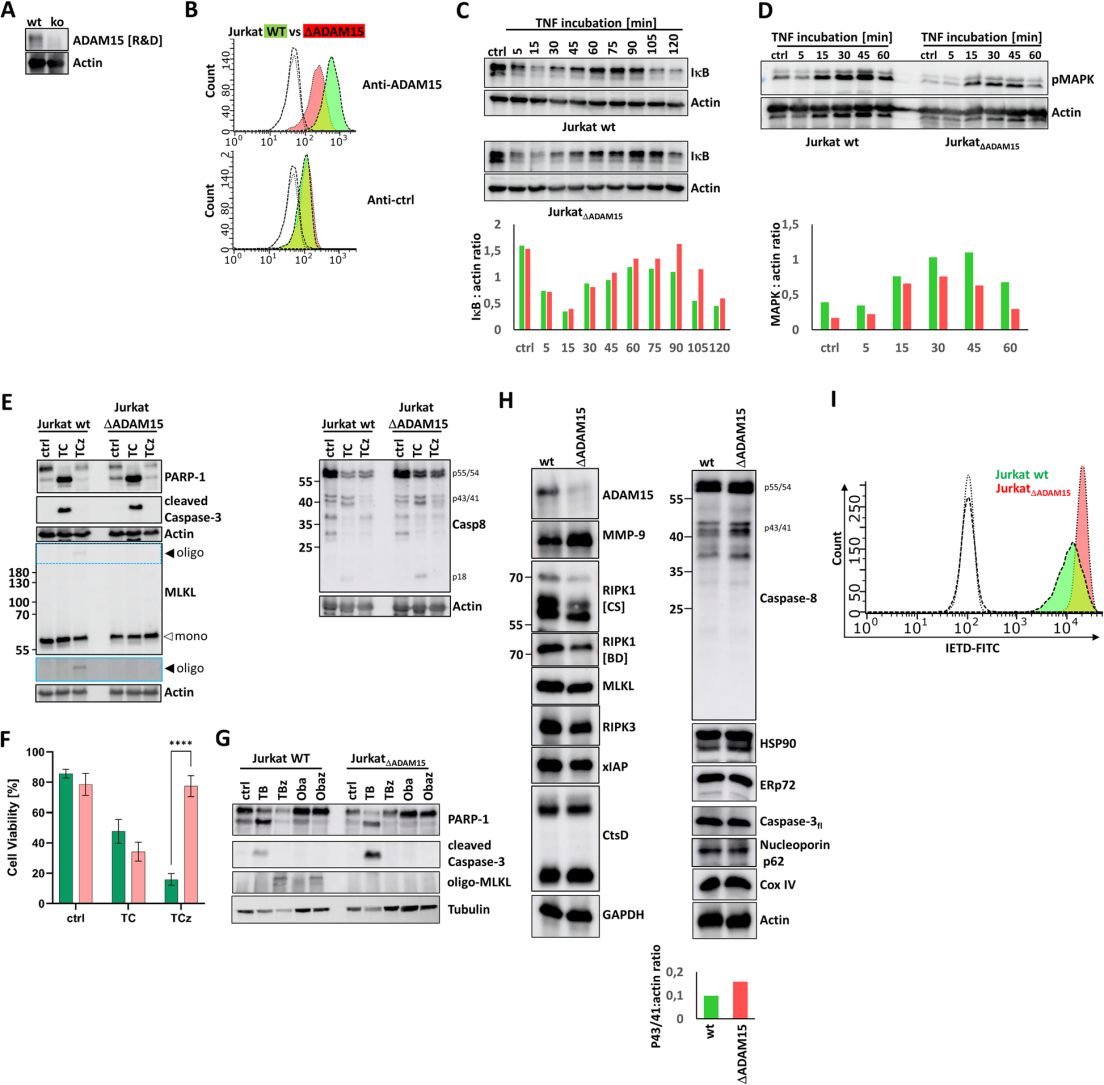
ADAM15 knockout in Jurkat cells results in abrogation of necroptosis induction and enhanced basal caspase-8 activity.** a** ADAM15 knockout was monitored by WB and** b** flow cytometry. The green curve represents staining using an anti-ADAM15 antibody (upper panel) or an isotype control (lower panel) in U937 wt cells. The red curve represents staining in Jurkat_ΔADAM15_ cells. The dashed non-coloured curves represent unstained cells. Right-shifted curves indicate higher fluorescence intensity. **c** degradation of IκB and **d** phosphorylation of MAPK are not affected by ADAM15 knockout. Band densities were quantified, and IκB:actin or MAPK:actin ratios are shown (green: Jurkat wt; red: Jurkat ΔADAM15). **e** WB analysis of cell death in Jurkat cells. **f** Representation of multiple (*N* = 4) flow cytometry-based cell death analyses (green bars: WT; red bars: ΔADAM15). **g** WB analysis of Obatoclax (Oba) mediated cell death in Jurkat cells. TNF/BV-6 or TNF/BV-6/zVAD treatment served as control. **h** WB was probed for cell death-related and unrelated proteins, revealing reduced amounts of full-length RIPK1 (third panel, left) and pre-activated caspase-8 (first panel, right). **i** The enhanced basal activation of caspase-8 was validated using flow cytometry. The dashed colorless curves indicate no staining. The green curve indicates wt cells stained with IETD-FITC, and the red curve indicates U937 ADAM15 cells with IETD-FITC. X-axis shift to the right side indicates increased fluorescence due to binding of IETD to active caspase-8 (p-18). In a, c, d, e, and f, actin or tubulin served as loading controls. Where not stated otherwise, representative data of n = 3 experiments are shown

To investigate whether ADAM15 loss also affects necroptosis in other cell types, we used CRISPR/Cas-9 to disrupt the *adam15* gene in Jurkat cells. Absence of the protein was verified by WB (Fig. 5A) and by flow cytometry (Fig. 5B). As observed for U937 cells, the kinetics of TNF-mediated NFκB and MAPK activation were not different in Jurkat wt compared to Jurkat_ΔADAM15_ cells (Figs. 5C and D).

Apoptosis induction occurred in both cell types, whereas necroptosis was absent in Jurkat_ΔADAM15_ cells upon stimulation with TNF (Fig. 5E and F) and Obatoclax (Fig. 5G).

As performed for U937 cells, band patterns in total lysates of Jurkat wt and Jurkat_ΔADAM15_ cells were analyzed by WB. Similar observations of altered RIPK1 and caspase-8 band patterns were made; however, they were not as pronounced as in U937 cells (Fig. 5H). Basal caspase-8 activity was moderately enhanced in ADAM15-lacking cells, as shown by flow cytometry (Fig. 5I).

Altogether, we show that ADAM15 plays a regulatory role in necroptosis induction across different cell types.

#### Expression of wt or L320A/D323N mutated RIPK1 did not restore necroptotic response

**Fig. 6:**
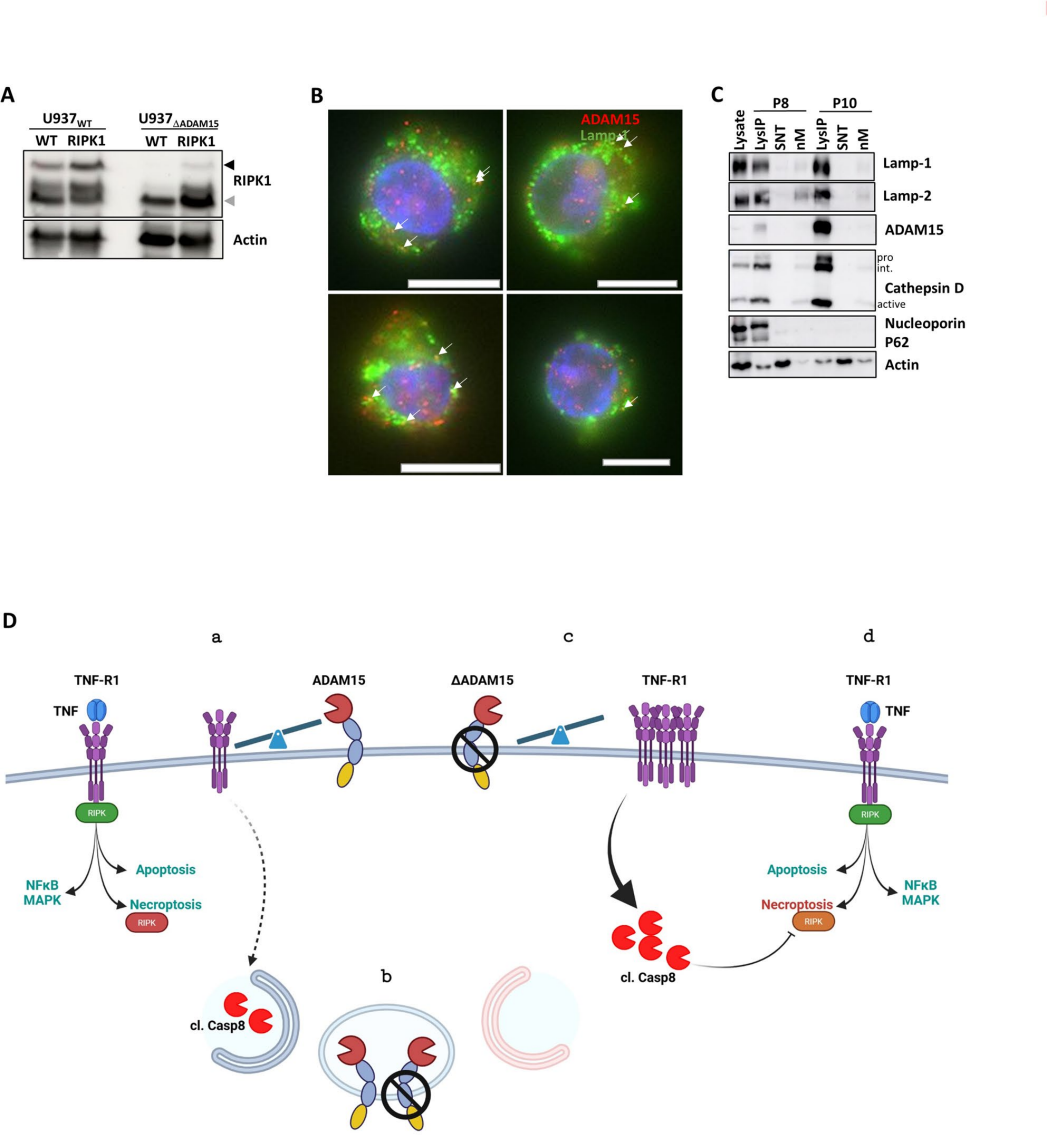
Exogenously expressed RIPK1 is degraded in U937_ΔADAM15_ cells, and ADAM15 is partially located in lysosome-like organelles. **a** WB analysis of exogenously expressed RIPK1: accumulation of full-length RIPK1 in U937 wt cells, and moderately in U937_ΔADAM15_ cells (black arrowhead). In U937_ΔADAM15_ cells, RIPK1 is directly cleaved (grey arrowhead). Actin serves as a loading control. **b** Lamp-2, Cathepsin D, and also ADAM15 are enriched in the lysosome-containing fraction (LysIP), compared to total lysate or soluble proteins (SNT). nM represents the lysosome-depleted non-magnetic fraction remaining after the isolation procedure. p8 and p10 represent two different homogenization procedures. Nucleoporin p62 and actin serve as loading/purity controls. **c** Fluorescence microscopy monitoring ADAM15 (red) and Lamp-1 (green) reveals partial co-localization (arrowheads) of both proteins in U937 cells. Manders’ tM1/tM2 values are: 0.495/0.619 (upper left); 0.403/: 0.362 (upper right), (lower left) 0.434/0.377; (lower right) 0.302/0.371 (lower right). Values of tM1/tM2 > 0.5 are considered moderate, > 0.7 strong colocalized. DNA (blue) is stained with DAPI. Scale bar indicates 10 µm. Where not stated otherwise, representative data of n = 3 experiments are shown. **d** Working model of ADAM15-mediated control of necroptosis induction (Model created in BioRender)

The degradation of RIPK1 by caspase-8, which thereby reduces necroptosis, was recently described [38, 55, 74]. We expressed wt RIPK1 in U937 wt and ΔADAM15 cells, which did not revert loss of necroptosis (not shown), but did show immediate partial degradation (Fig. 6A). Next, we tried to express L320A/D323N double-mutated RIPK1, which, however, was toxic for the cells.

#### ADAM15 is located in intracellular vesicular compartments

While the loss of ADAM15 induced moderate caspase-8 activation and RIPK1 degradation, the underlying mechanism is unclear. Analyzing possible regulated pathways by GSEA upon ADAM15 loss, we identified protein groups involved in regulating membrane/vesicle fusion processes. In line, we recently reported detoxification of TNF-induced low levels of active caspase-8 via an unconventional ATG9a-mediated lysosomal detoxification pathway [30]. Following this, we performed fluorescence microscopy and observed intracellular ADAM15 protein and partial co-localization with the lysosomal marker protein lamp-1 (Fig. 6B). To verify co-localization in the same compartment, we used subcellular organelle fractionation via lamp-1 pull-down [25]. Probing the derived fractions for lysosomal markers (Lamp-1, −2, and Cathepsin D), we showed that these are enriched. Using a mild homogenization scheme that does not fully detach intracellular organelles and membranes, we observe nucleoporin p62 as “nuclear contamination” and proportionally less ADAM15. As observed before, harsher homogenization (P10) results in a higher purity of lysosomes and proportionally more ADAM15 (Fig. 6C). Reciprocal isolation using the available ADAM15 antibodies was not possible, as both MAB935 and HPA011633 bind the ectodomain, which is located inside a vesicle. However, such an approach would enable us to define the intracellular and vesicular role of ADAM15 and investigate its catalytic activity in this compartment.

## Discussion

Initially described as an accidental, lytic, and highly pro-inflammatory type of cell death, necrotic cell death was found to be tightly regulated, hence termed necroptosis. Understanding its molecular regulation may lead to novel treatment options by triggering immunogenic cell death in cancer or reducing tissue damage following infection or transplantation [50]. Activating death receptors, such as TNF-R1, TRAIL-Rs, CD95/Fas, or DR3, can trigger regulated necroptosis, with necrosome-forming proteins like RIPK1, RIPK3, and MLKL serving as major regulators. It was recently demonstrated that, once triggered, cells can prevent full necroptotic death by capturing MLKL pores from the plasma membrane through an ESCRT-mediated process [27].

Here, we unveil ADAM15 as a novel player in the cell death network. ADAM15 was initially identified and cloned as ‘metargidin’ in a PCR screen for ADAM proteases [37]. It comprises all protein domains present in other ADAM proteases but contains a so-called RGD domain, making it unique among its family members. RGD domains have been described in various animal toxins and confer the ability to bind integrins, i.e. αvβ3 or α5β1 [44, 45].

Accumulating evidence shows that S-acylation regulates apoptotic and non-apoptotic cell death [34, 47]. ADAM15 was suggested to be post-translationally modified in proteome screens for (differentially) S-acylated proteins in different types of cells and organisms [49, 52, 90]; two studies involved the analysis of cell death induction [59, 62]. The protein possesses possible palmitoylation acceptor cysteine residues (Cys726, Cys733, and Cys830; however, formal proof of ADAM15 S-acylation and corresponding regulatory roles is lacking. In addition, possible palmitoyl transferases or thioesterases remain to be identified.

Enhanced ADAM15 expression has been described for many cancer types and correlates with poor prognosis and metastasis formation [5, 21, 43, 60, 62, 63, 83]. However, data from colon cancer studies have shown that ADAM15 overexpression inhibits metastasis formation, suggesting different roles for ADAM15 in other tissues [75].

Expression of ADAM15 in cells (e.g., U937 or Jurkat) derived from hematologic diseases has been reported, while biological functions have not been investigated in depth [81]. So far, most functions of ADAM15 in intestinal bowel disease, cardiac disease, atherosclerosis, rheumatoid arthritis, lung diseases, angiogenesis, and cancer formation have been attributed to its plasma membrane-resident form in adherent cells of different origins. It is assumed that ADAM15 primarily acts by mediating the ectodomain shedding and release of signaling molecules, facilitating cell–cell interactions, ECM remodeling, and cell migration. However, only a limited number of substrates have been identified, and it was shown that ADAM15 functions independently of its catalytic activity, e.g., by regulating the mRNA level of proteins [85]. We observed enhanced surface expression of the death receptor TNF-R1, which was accompanied by elevated protein levels. Such increased surface expression may trigger the ligand-independent clustering of surface receptors via aggregation of their intracellular death domains, in membrane regions referred to as CASMER (cluster of apoptotic signaling molecule-enriched rafts), possibly internalization, and can result in the activation of caspase-8 [2, 5, 26, 54]. However, it has to be proven in future studies that TNF-R1 is a *bona fide* ADAM15 substrate. Alternatively, other mechanisms like post-translational modification (e.g., palmitoylation) and the recruitment to specialized membrane domains could be involved [24].

Approximately half of the ADAM15 protein is localized intracellularly in endosomes and trans-Golgi vesicles, and thus is likely to have hitherto unknown intracellular functions [46]. Vesicle-located ADAM15 is likely the source of ADAM15-containing extracellular vesicles, which have cancer-modulating activity [40]. The intracellular domain of ADAM15 contains potential phosphorylation sites and proline-rich sequences, which facilitate interaction with Src homology 3 (SH3) domain-containing proteins (i.e., Lck, Hck, Grb2), suggesting regulation of intracellular signaling [57]. ADAM15 activity was functionally linked to regulating FasL-mediated apoptosis in synovial cells/tissue by activating the Src/FAK pathway [5, 33].

We observed intracellular ADAM15, which is partially co-localized with Lamp1 and Lamp2-positive vesicles. As there is no complete overlap, we postulate an ‘ADAM15-some’ for which the molecular composition, e.g., whether these include caspase-8 or RIPK1, has to be characterized in detail. GSEA analyses based on the proteome analysis suggest that ADAM15 regulates membrane/organelle trafficking and fusion processes.

Endocytosis and vesicle/autophagosome fusion were among the major regulated cellular processes when comparing wt and ADAM15-lacking cells. This reorganization of intracellular organelle trafficking remains to be formally proven; however, it may represent a means to modulate necroptosis, e.g., by detoxification of MLKL pores as reported by Gong and colleagues [27]. A recent report on tick-borne encephalitis virus (TBEV) revealed that the loss of ADAM15 impacts the U251 cellular endomembrane system, thereby affecting virus replication. On the other hand, ADAM15 subcellular localization is altered upon TBEV infection [86]. Liu and colleagues recently showed that ADAM15, 10, and 17 regulate mTORC1 and thus autophagy in Triple-negative breast cancer (TNBC) cells [42].

Optineurin (Optn), another protein frequently associated with necroptosis regulation, displayed increased expression in ΔADAM15 cells. It directly binds to and regulates the function of caspase-8 [15, 31, 62], of proteasome-mediated RIPK1 stability in amyotrophic lateral sclerosis [31], as well as vesicle trafficking and autophagy, and therefore represents a promising regulator of vesicular ADAM15 [35, 53]. In a recent study, ADAM15 was linked to aggrephagy, an autophagy-related process that regulates cancer progression [82].

The extent of such changes in organelle organization and trafficking has to be investigated in greater detail in the future. Especially if ADAM15 catalytic activity (inside the compartment) is required to regulate necroptotic cascades in a similar manner.

While we observed partial proteolytic processing of RIPK1, both TNF-induced apoptosis induction and activation of NFκB were functional, revealing IκB degradation in the same time frame. However, the IκB signal in ADAM15-lacking cells initially was and remained slightly stronger, which may be explained by the central function of RIPK1 in TNF-mediated *complex I* formation and NFκB activation. The overall RIPK1 protein level was not altered in our proteome screen. This implies that separate pools of RIPK1 might reside in different intracellular compartments (cytosolic or plasma membrane resident), potentially shielding them from or exposing them to caspase-8-mediated cleavage. Such compartmentalization could also be regulated by S-acylation, as it was recently reported that TNF triggers ubiquitination-dependent and zDHHC5-mediated RIPK1 S-acylation as a pre-requisite for cell death induction [89]. Also, for active caspase-8, such compartmentalization has been shown: active caspase-8 can be TNF-R1-bound or part of TNF-R1 dissociated *complex II* [20, 51, 65], aside from localization at the plasma membrane or in the cytoplasm [4, 36]. Differential subcellular localization of proteins selectively enables or disables them to interact with, e.g., substrates.

Another protein that interacts with and stabilizes RIPK1 is the mitochondrial serine proteinase HTRA2 [88]. Depletion or inhibition of HTRA2 protects from TNF-induced necroptosis [69, 70]. In our screen, HTRA2 is enriched in wt cells and grouped into the ‘regulation of autophagy’ group by GSEA.

We found the LUBAC component HOIL1 enriched in wild-type cells. LUBAC is a regulator of TNF-mediated cell survival [28], but loss of LUBAC activity has also been shown to result in reduced TNF-mediated necroptosis, recruitment of active MLKL to membranes is inhibited [77].

Aside from DR-mediated necroptosis, we observed the absence of Obatoclax-mediated necroptosis in U937_ΔADAM15_ cells. Previous reports showed that Obatoclax-mediated necroptosis involves the formation of the necrosome on autophagosomal membranes in different cell types [3, 5, 72].

Further GO-functional groups modulated upon ADAM15 loss were the regulation of cell redox homeostasis, epigenetic gene regulation, and mitotic cell cycle transition. The latter was investigated in NSCLC cells, where ADAM15 is upregulated and knockdown increases G0/G1 phase cells [62]. Also, in TNBC cells, knockdown of ADAM proteases, including ADAM15, reduces cell proliferation and growth [42]. Tryptase, a protein we found to be enriched in ADAM15-lacking cells, regulates histone modification. Inhibition of tryptase results in enhanced necrotic death in HMC-1 cells [1].

Taken together, we propose the following model (Fig. 6D): a) Under wildtype conditions, ADAM15 regulates the steady state surface expression of TNF-R1 (and DR4), which allows for functional induction of NFκB/MAPK, apoptosis, and necroptosis induction. b) In case minor amounts of active caspase-8 are produced (e.g., due to altered receptor clustering), these molecules are detoxified by autophagy. c) In the case of lacking ADAM15, surface expression of TNF-R1 is enhanced, resulting in low levels of falsely activated caspase-8, which cannot be detoxified, as the absence of ADAM15 also affects vesicular/autophagic organelle organization. d) activated caspase-8 partially degrades RIPK1, still allowing for NFκB or apoptosis but not for necroptotic signaling induced by death receptors or other factors.

### Limitations of the study

The role of ADAM15 in regulating necroptosis needs to be verified in additional cell lines, primary cells, and multicellular organisms. For example, we previously observed differential regulatory roles for the ADAM17 protease in endothelial versus blood cell lines [5, 23]. The heterogeneity of amino acids may affect post-translational modification and suggests different modes of action in other organisms. Additionally, the expression of different splice variants may impact ADAM15's function as a regulatory molecule.

The relevance of putative ADAM15 palmitoylation and its catalytic activity (e.g., for TNF-R1 shedding) in the process of regulating necroptosis remains to be further investigated.

We observed differentially regulated protein networks upon ADAM15 knockout, especially many affecting organelle regulation and trafficking. This, and its functional impact, need to be further analyzed. It must be demonstrated whether any of the proteins involved in the networks are involved in, for example, the incorporation of RIPK1 into a vesicle for degradation or the exclusion of active caspase-8 from the vesicles. It must also be investigated to what extent up- or downmodulation of such proteins phenocopies loss of ADAM15.

The expression of mutant, cleavage-resistant RIPK1 was lethal in cells, likely due to the promotion of RIPK1-dependent cell death [74]. Thus, it has to be proven that ADAM15-induced activation of caspase-8 results in partial RIPK1 cleavage and, therefore, abrogation of necroptosis.

Experiments using exogenous expression (mutated) ADAM15 have to be performed to decipher which ADAM15 functional or scaffolding domains are involved in the regulation of necroptotic death. Similarly, it could be interesting to design ADAM15-specific inhibitors to test their effect on necroptosis induction.

Our observations align with earlier findings that proteins can have opposed functions when located in different subcellular compartments. Similarly, we observed intracellular ADAM15 in different vesicular compartments. To resolve this, high-resolution spatiotemporal imaging, subcellular fractionation, and organelle characterization are required to decipher which reactions occur in which compartment.

## Conclusion

While the precise molecular mechanism by which ADAM15 regulates caspase-8 activity and RIPK1 cleavage remains unclear, ADAM15 is a novel regulator of extrinsic and intrinsic necroptosis. Genetic ablation elevates TNF-R1 surface expression, affects several cellular pathways, including organelle fusion, trafficking, or autophagy, and activates partial RIPK1 cleavage by caspase-8. Our findings highlight the partitioning of total cellular proteins into distinct compartments, which is crucial for mediating various biological outcomes, including cell death and survival. Future research should focus on the subcellular compartmentalization of multiple pools of ADAM15 molecules, their potential differential and dynamic post-translational modification states, and thus the regulation of cell death processes. A comprehensive understanding of the diverse cellular roles of ADAM15 may enable its therapeutic exploitation in the future for inflammatory, infectious, or cancer-related diseases.

## Supplementary Information

Supplementary Material 1.

## Data Availability

The mass spectrometry proteomics data have been deposited to the ProteomeXchange Consortium (https://proteomecentral.proteomexchange.org) via the jPOST partner repository [56] with the dataset identifiers PXD066056 (ProteomeXchange) and JPST003922 (jPOST). To review the data: Go to: [https://repository.jpostdb.org/preview/300089052687116bd50a22], (https://repository.jpostdb.org/preview/300089052687116bd50a22) Access key: 4853 All source code and data files are available from the authors upon request.

## Abbreviations

7AAD: 7-Aminoactinomycin D
AF: Alexa-Fluor
ADAM15/17: A disintegrin and metalloproteinase 15 / 17
CHX: Cycloheximide
DR: Death receptor
FITC: Fluorescein isothiocyanate
GSEA: Gene set enrichment analysis
IAP: Inhibitors of apoptosis protein
LUBAC: Linear ubiquitin chain assembly complex
MAPK: Mitogen-activated protein kinase
MLKL: Mixed lineage kinase domain-like pseudokinase
MS: Mass spectrometry
MOMP: Mitochondrial outer membrane permeabilization
NFκB: Nuclear factor kappa-light-chain-enhancer of activated B cells
RIPK1/3: Receptor-interacting protein kinase 1 / 3
SMAC: Second mitochondria-derived activator of caspases
TL1a(-R): TNF like cytokine 1a(-receptor)
TNF(-R1): Tumor necrosis factor (receptor 1)
TRAIL(-R): TNF related apoptosis inducing ligand (-receptor)
WB: Western blot
wt: Wild type
zVAD: Carbobenzoxy-valyl-alanyl-aspartyl-[O-methyl]-fluoromethylketon
