# Supplementary figures and images for: Loss of ADAM15 prevents necroptosis induction by partial RIPK1 degradation due to enhanced TNF-R1 surface expression and basal caspase-8 activation
Source: Cell Commun Signal. 2025 Dec 4;23:520. doi: 10.1186/s12964-025-02530-3 (PMC12676761; doi:10.1186/s12964-025-02530-3)

A

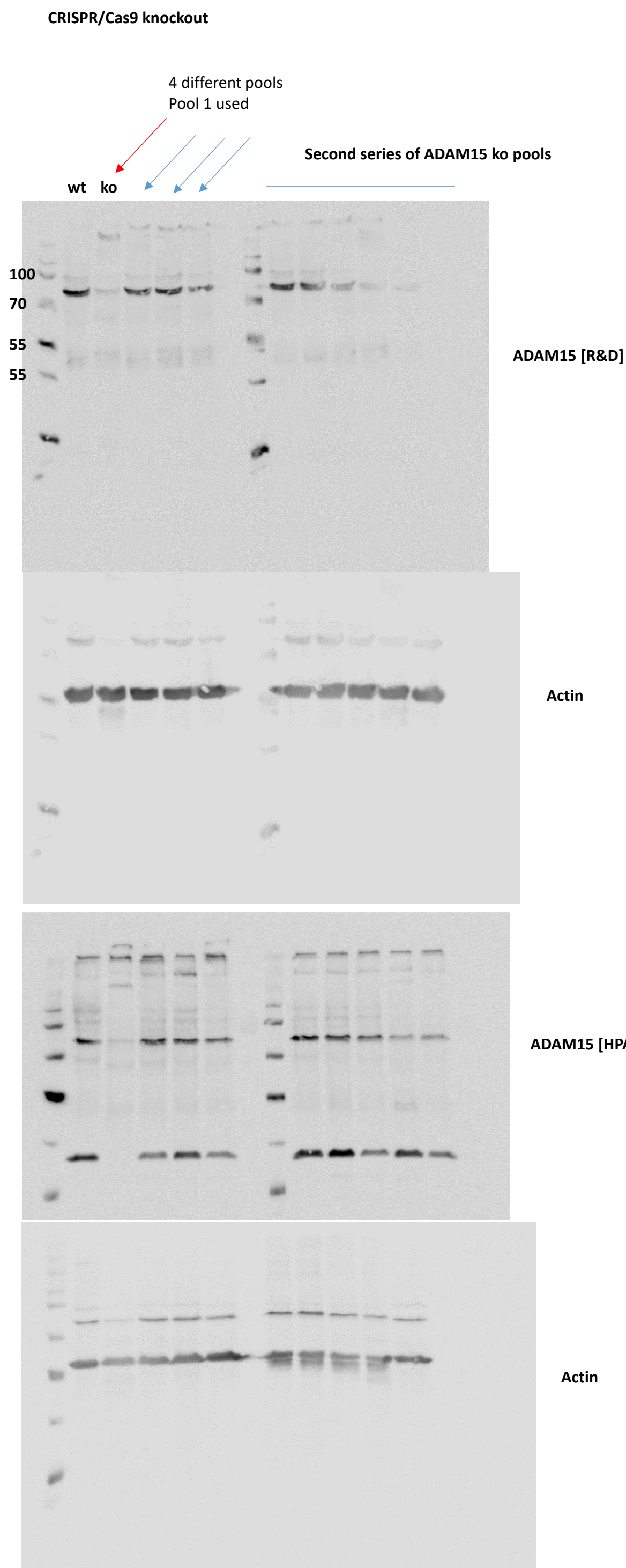

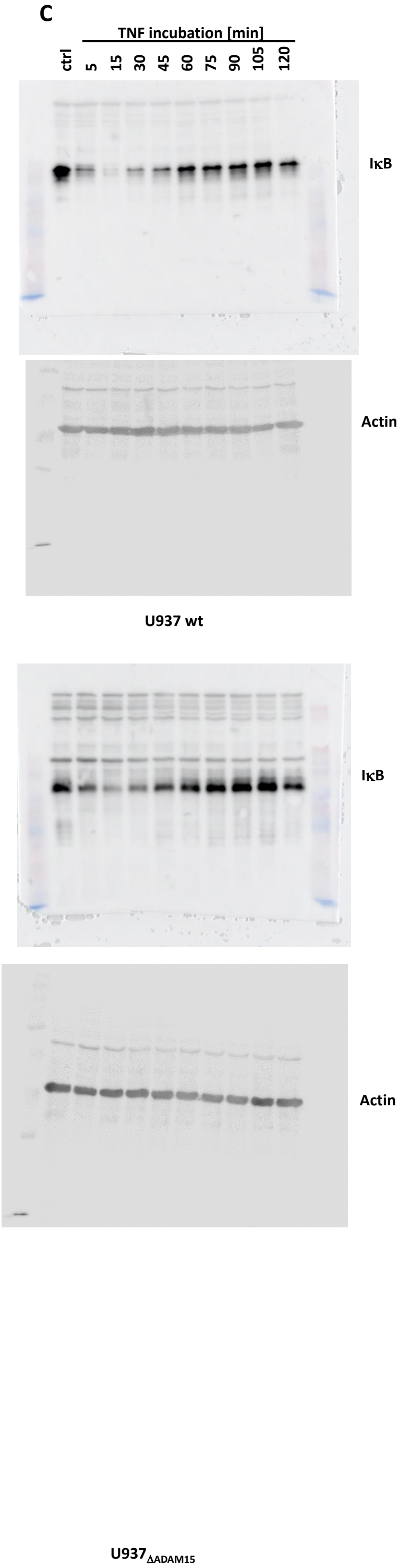

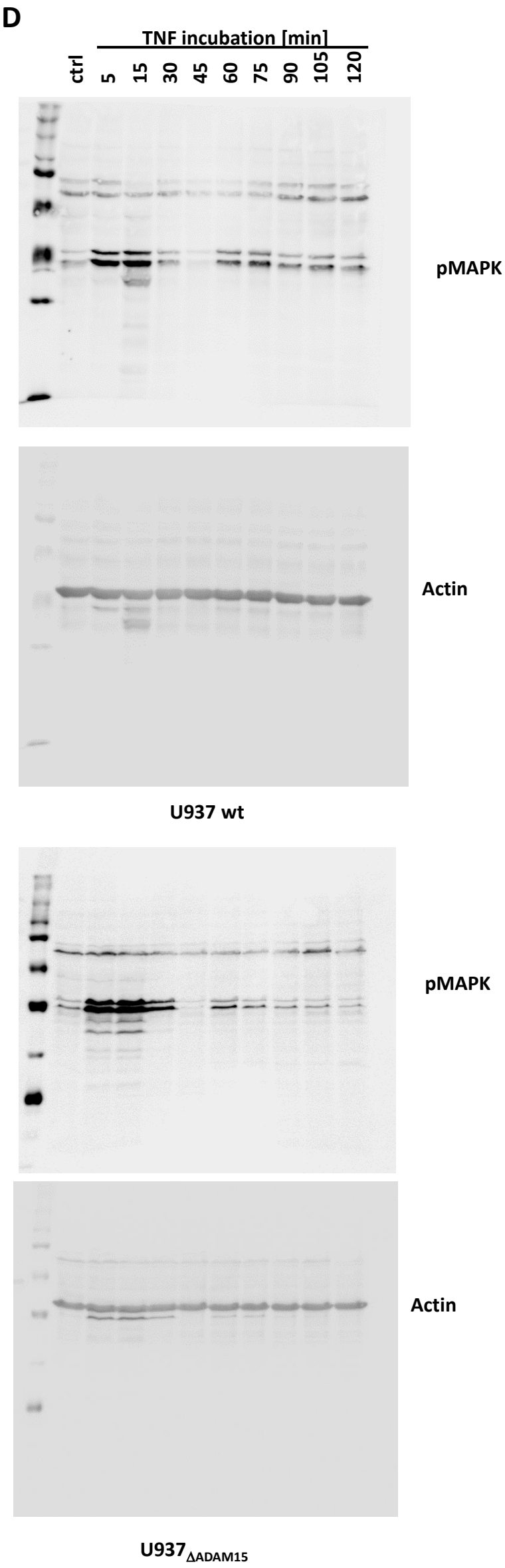

**F**

**Figure 1**

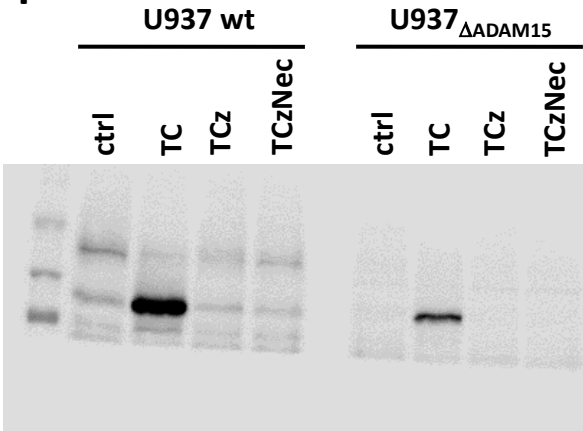

PARP1

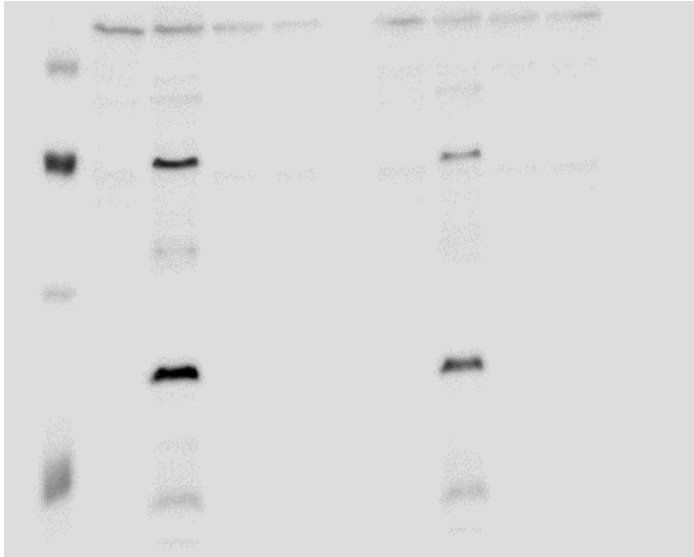

Cleaved Caspase-3

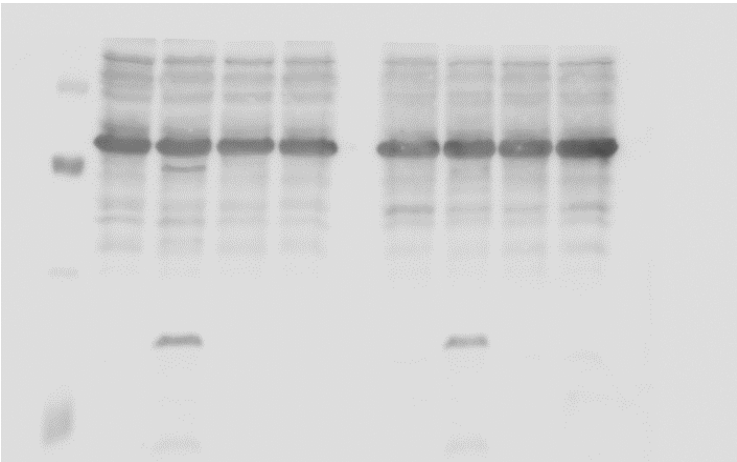

Actin

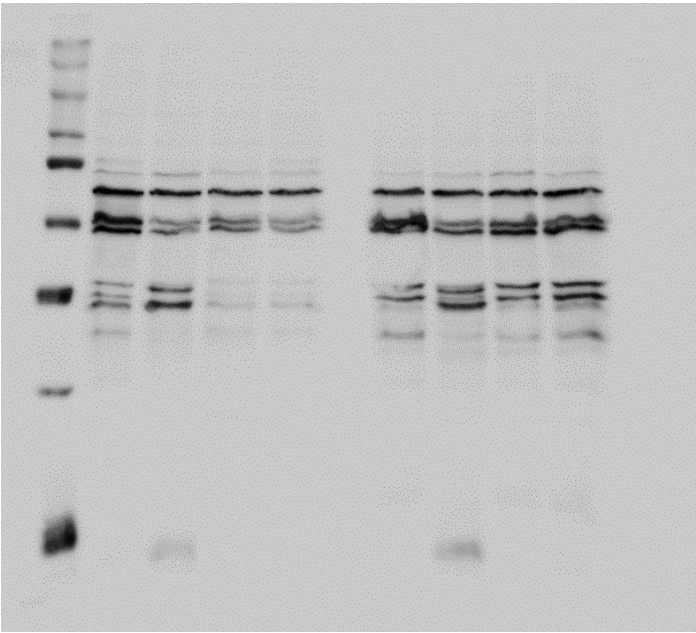

Cleaved Caspase-8

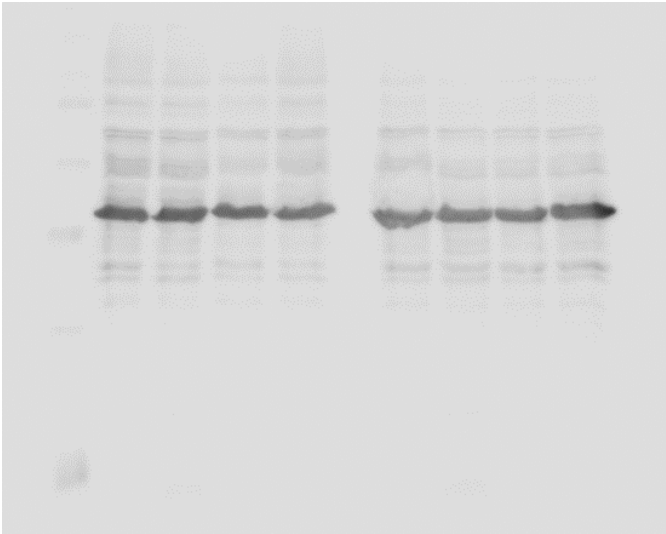

Actin

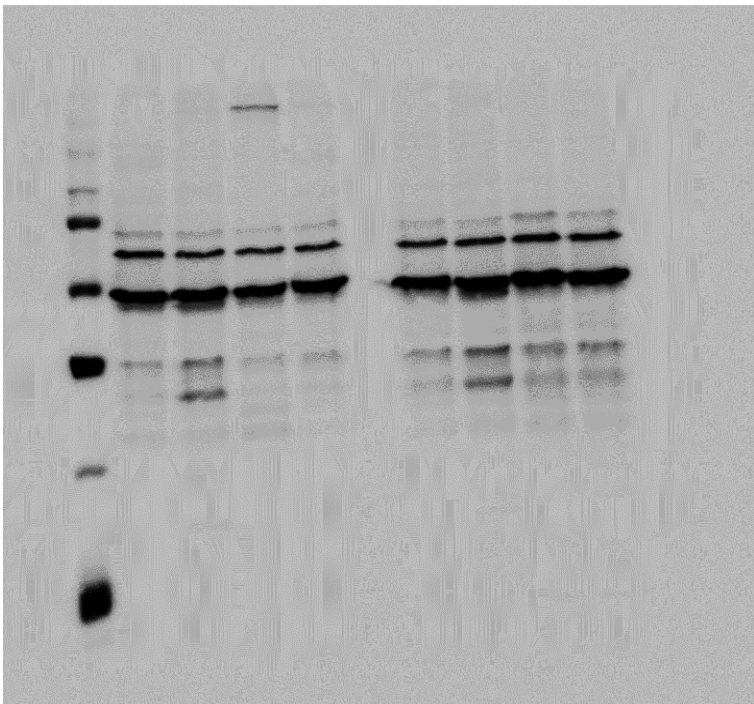

oligo

MLKL

mono

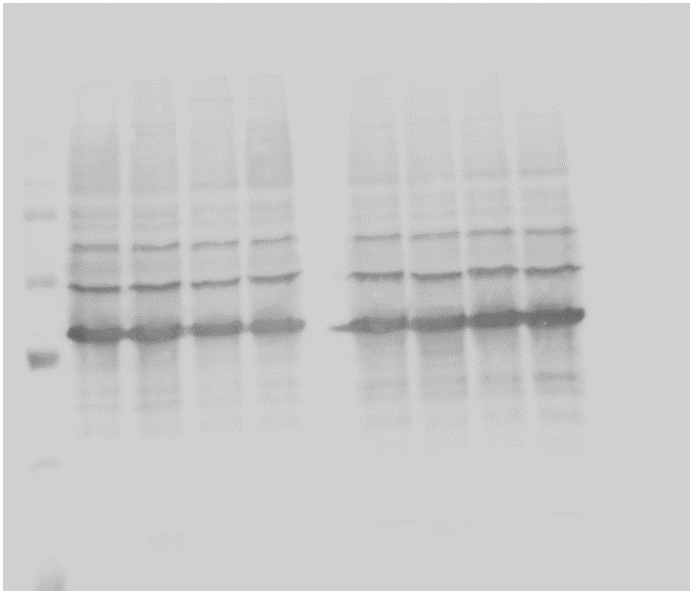

Actin

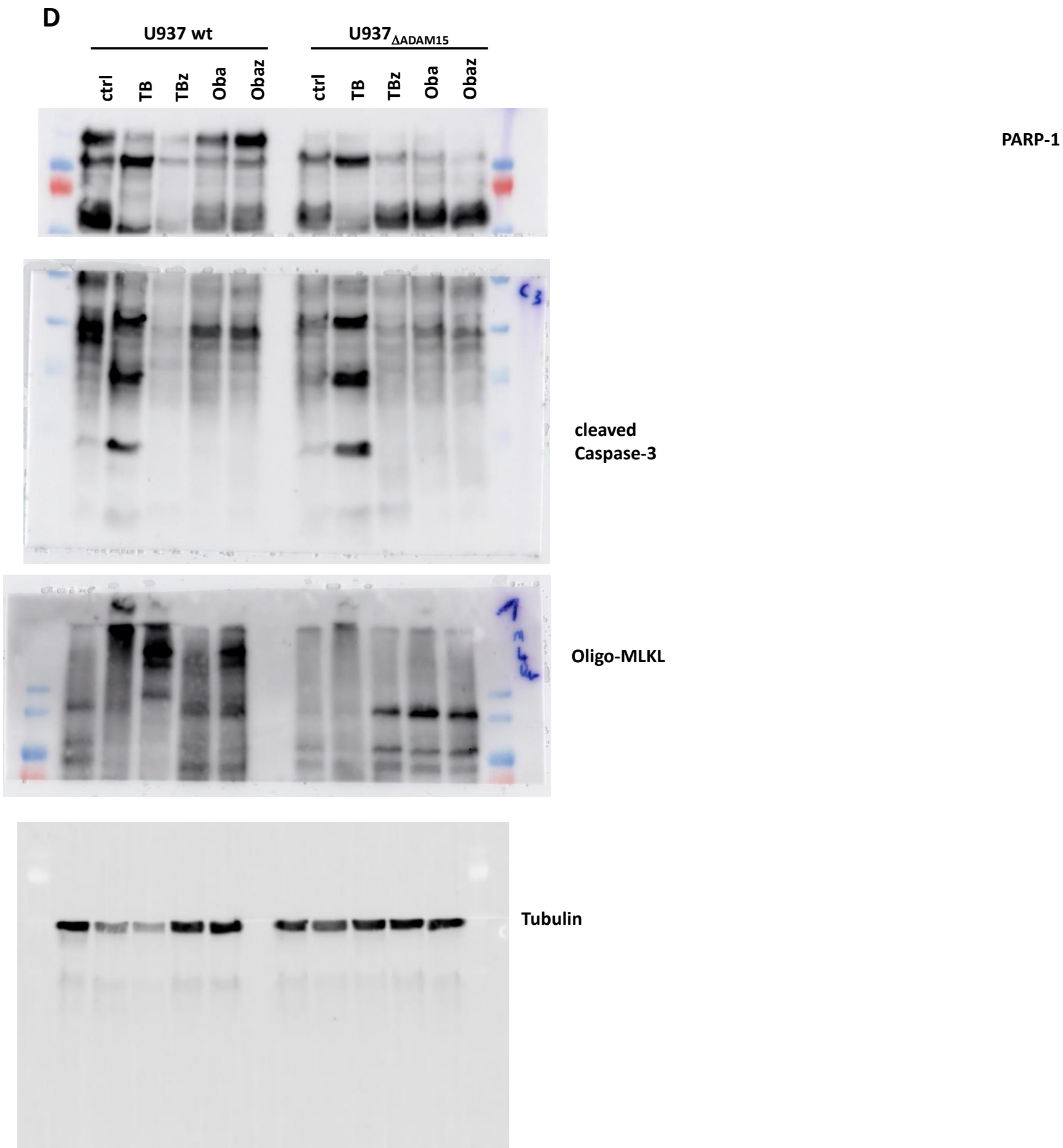

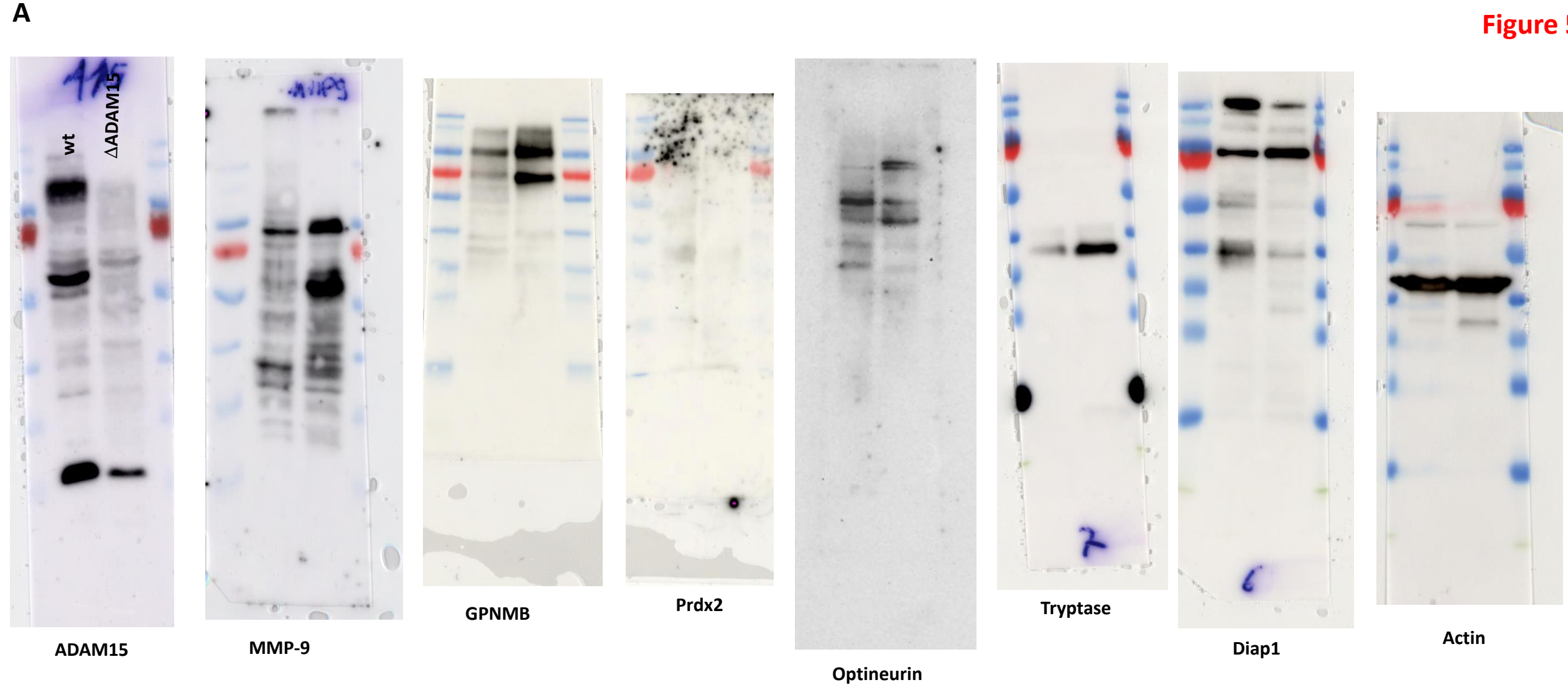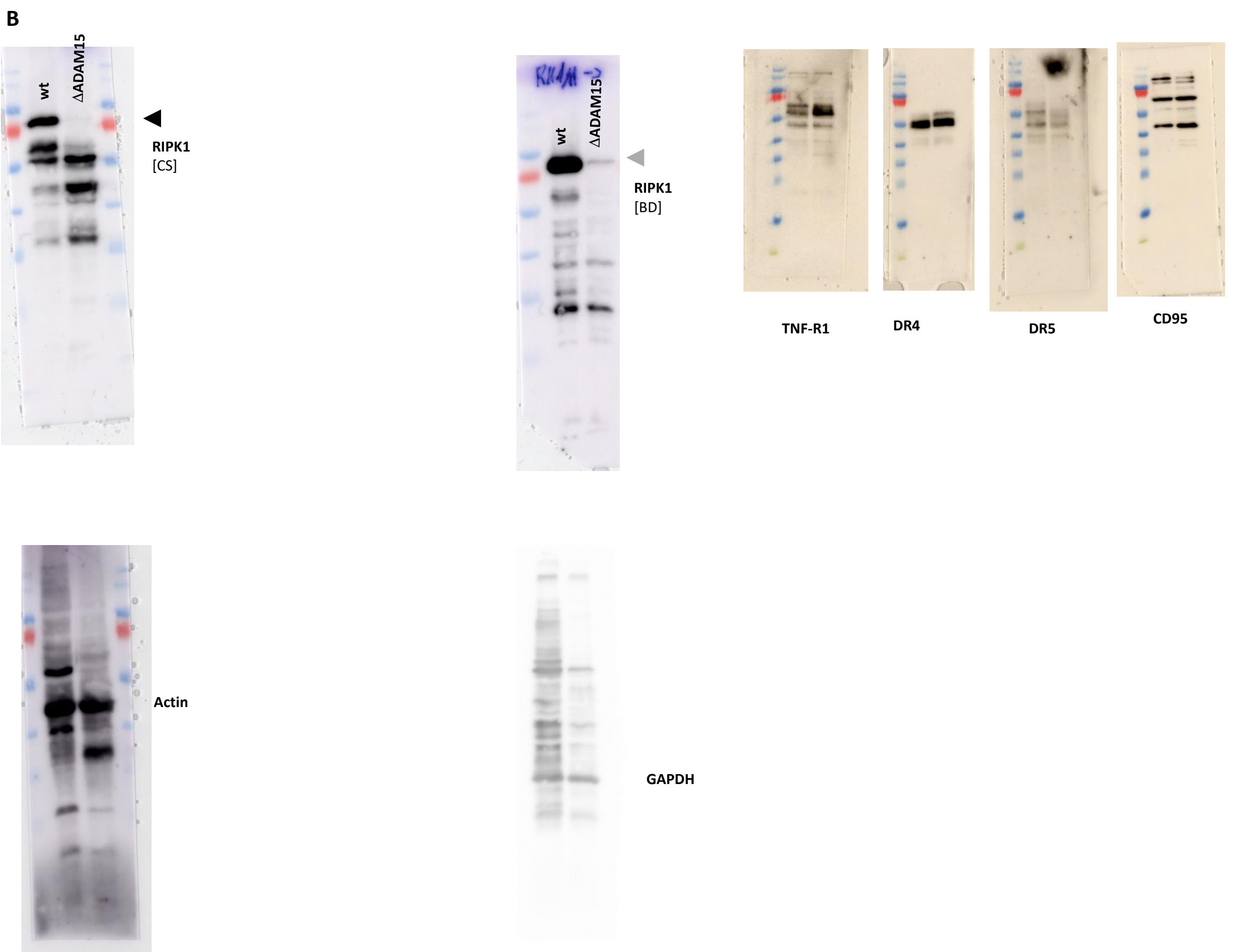

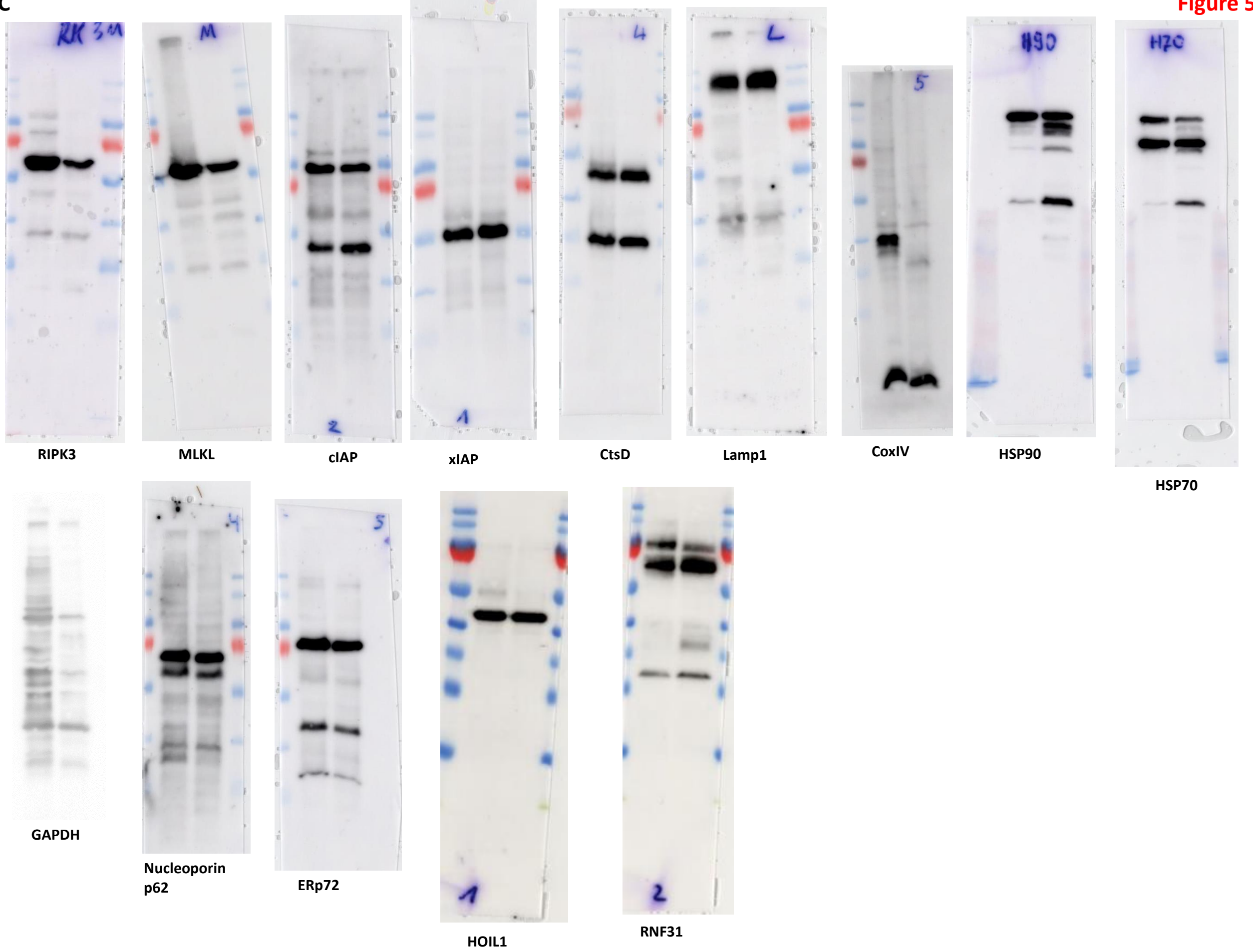

D

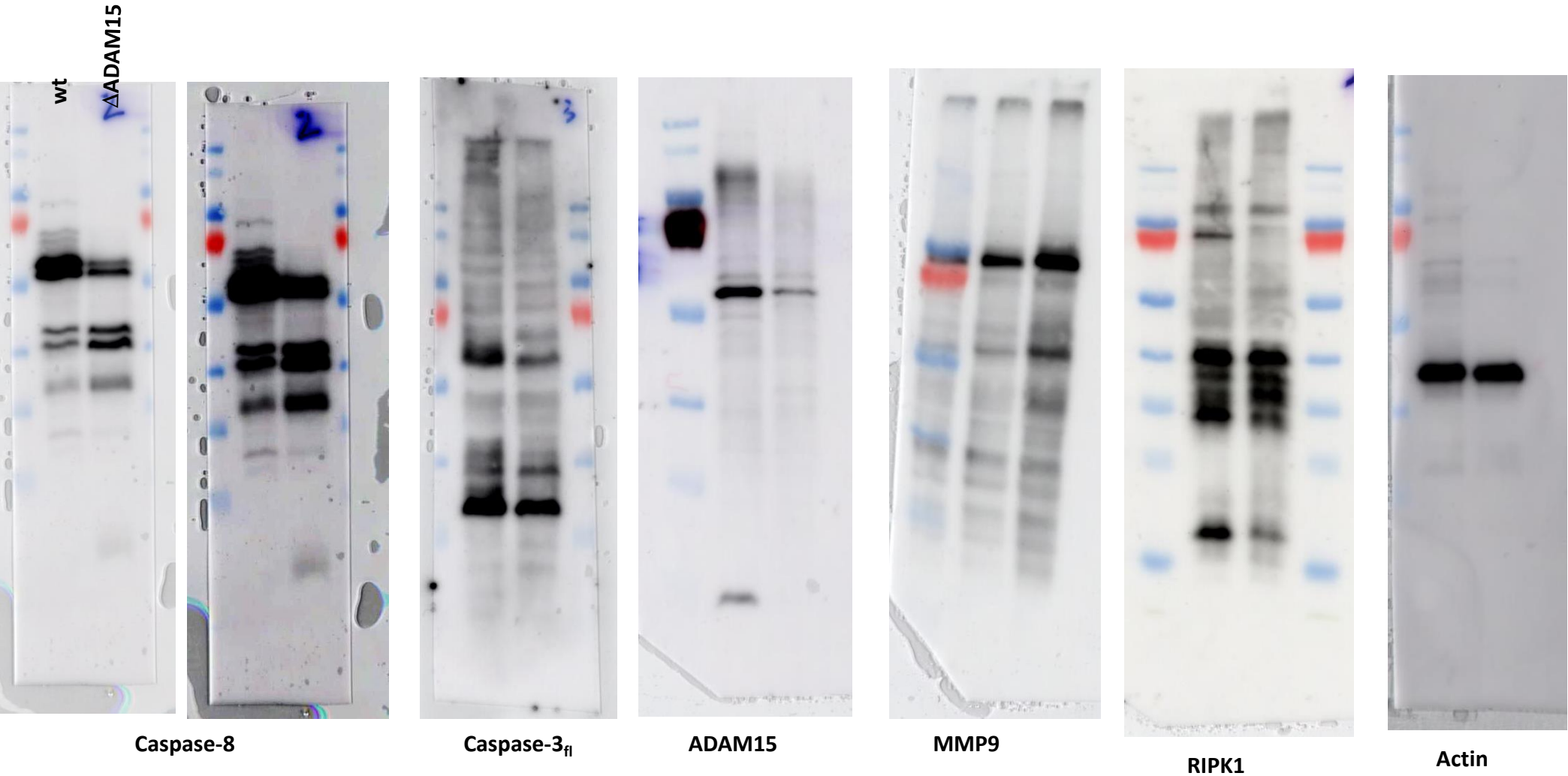

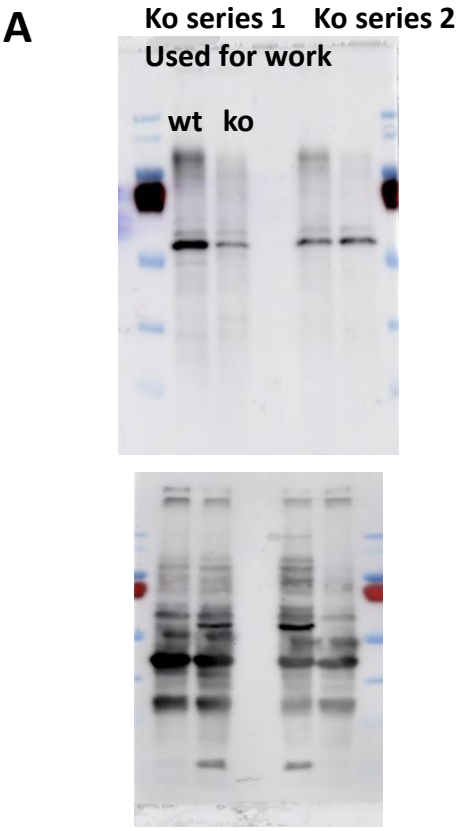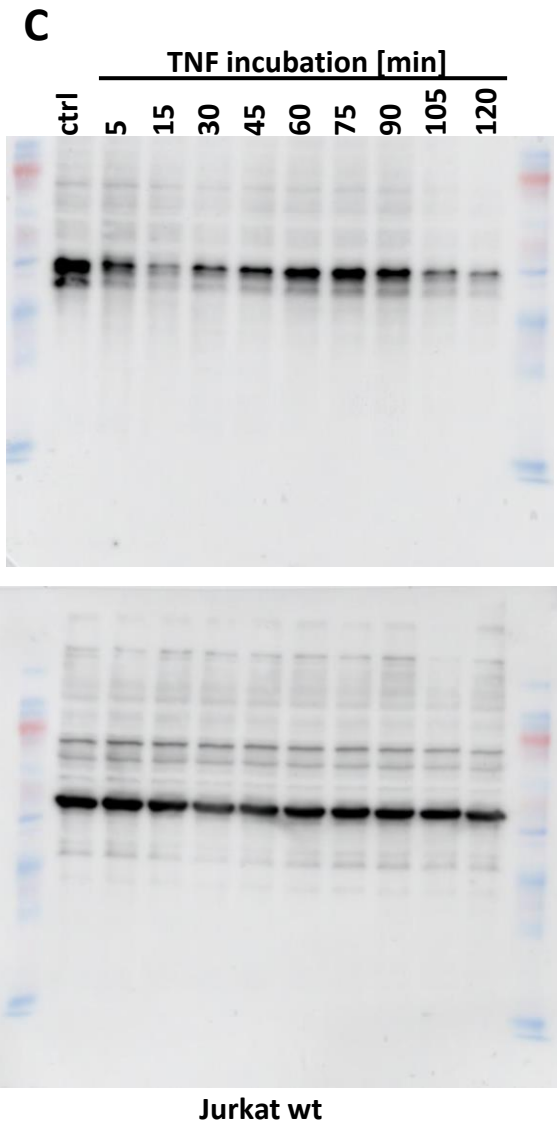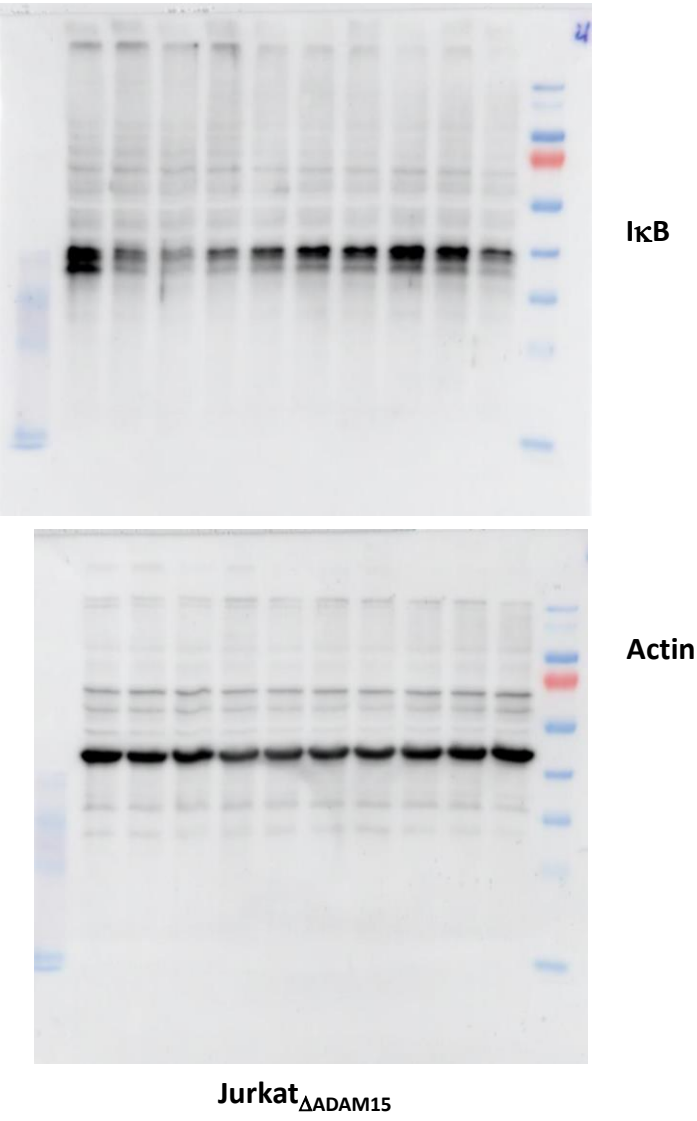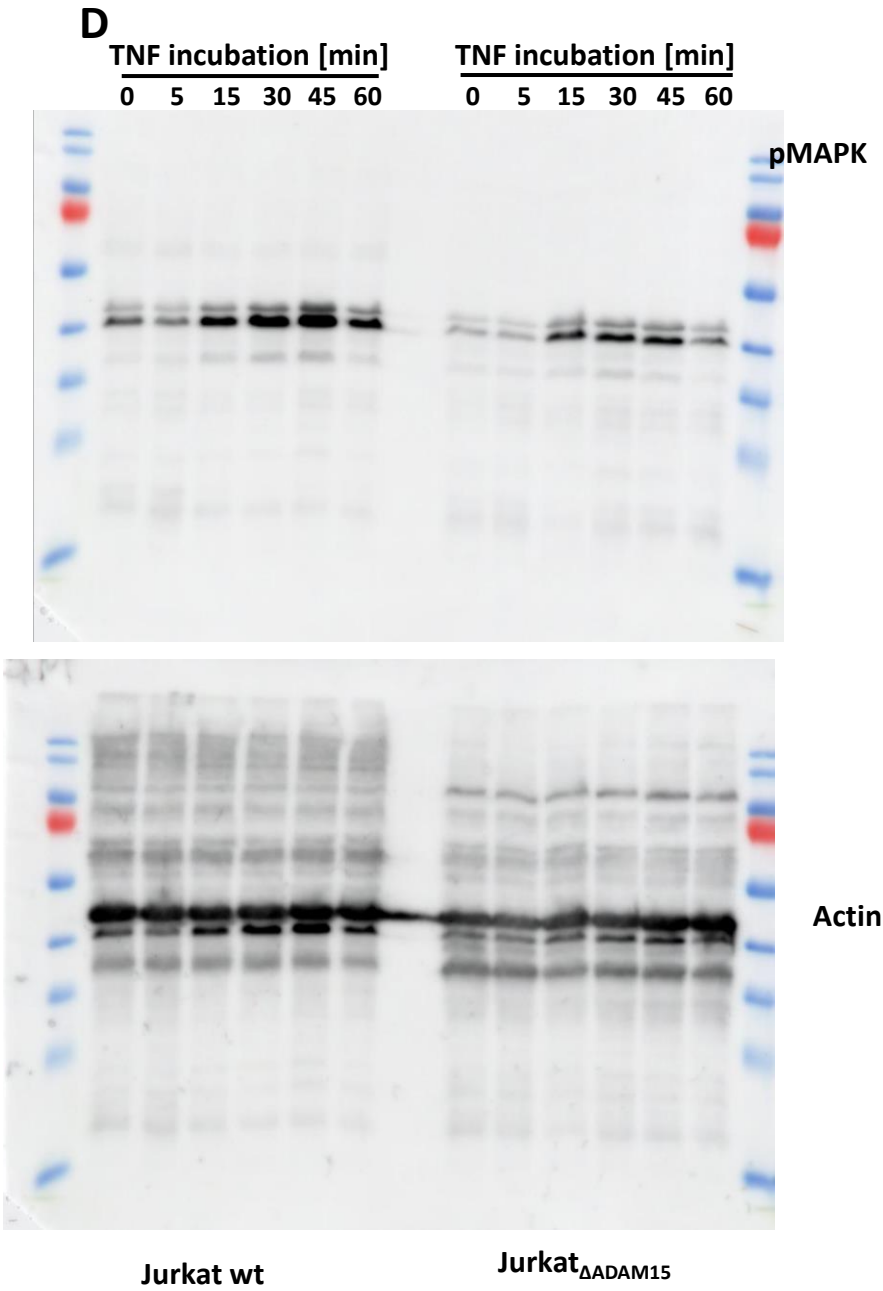

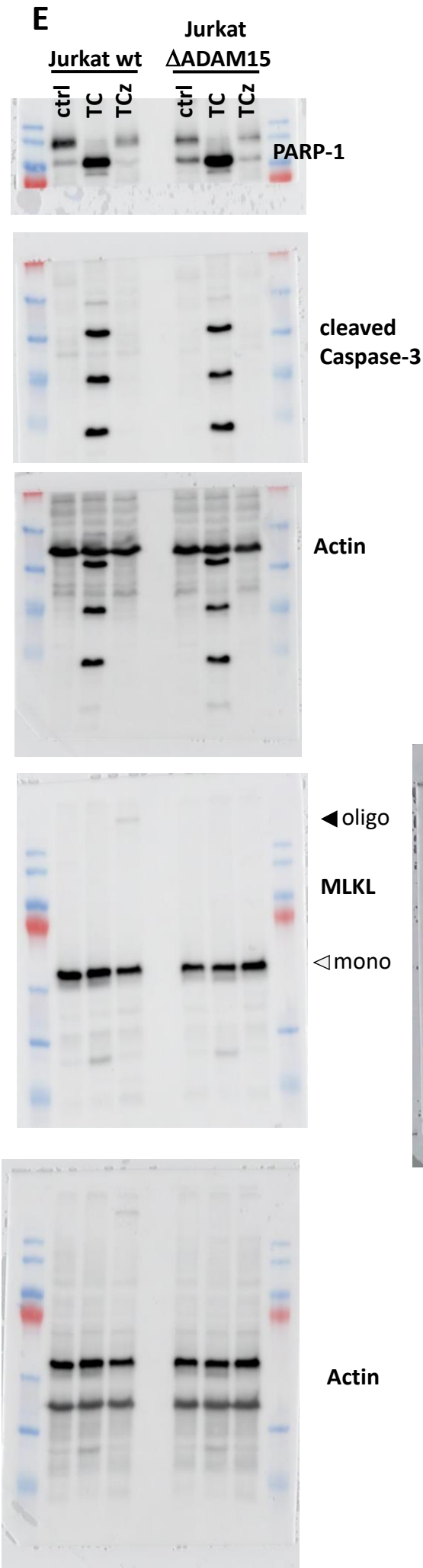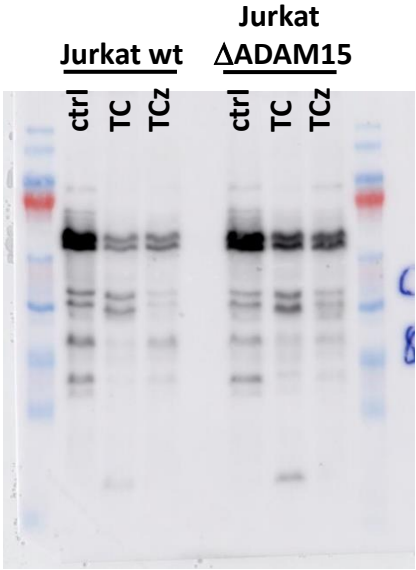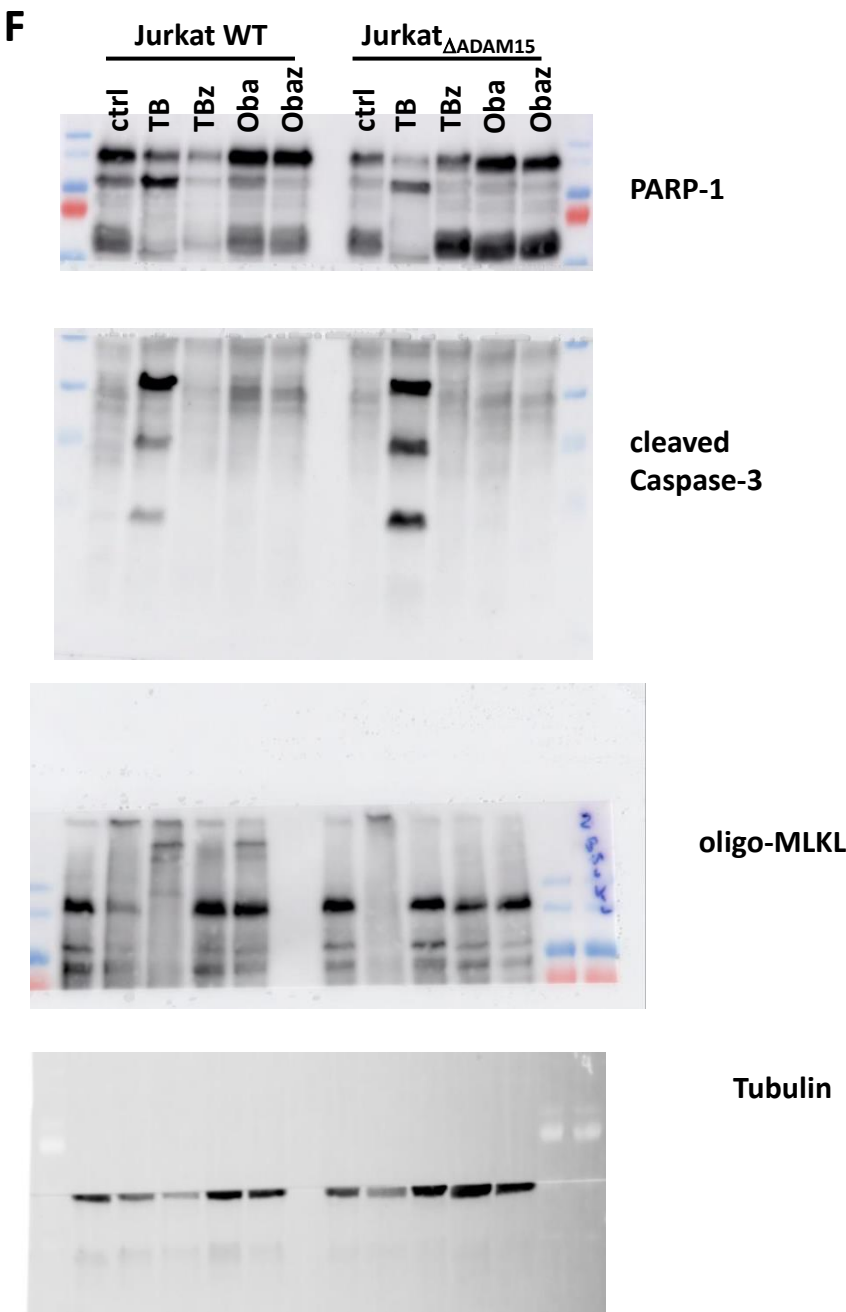

G

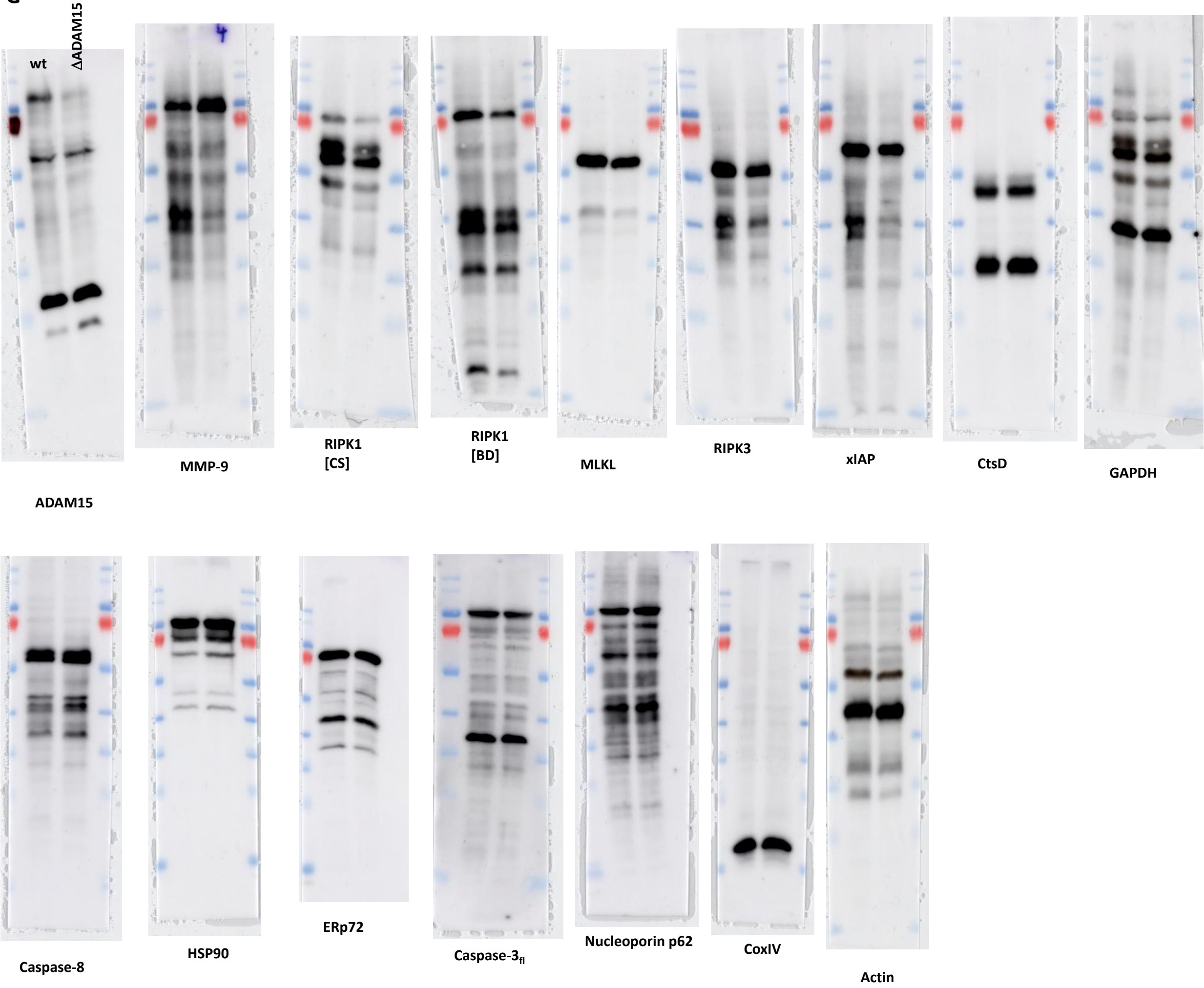

A

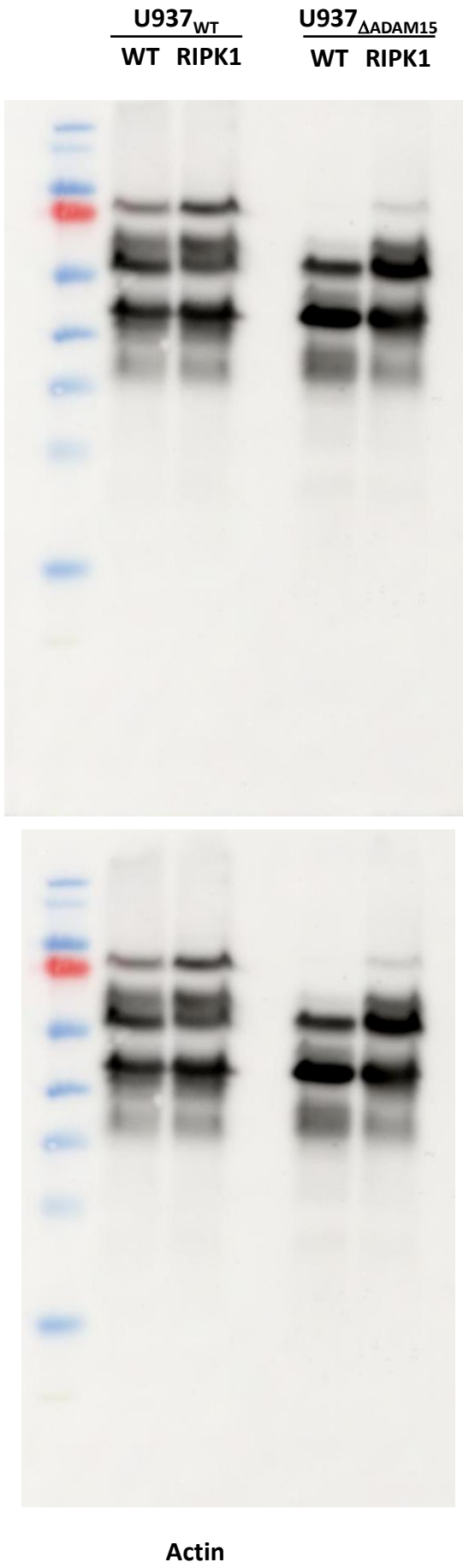

B

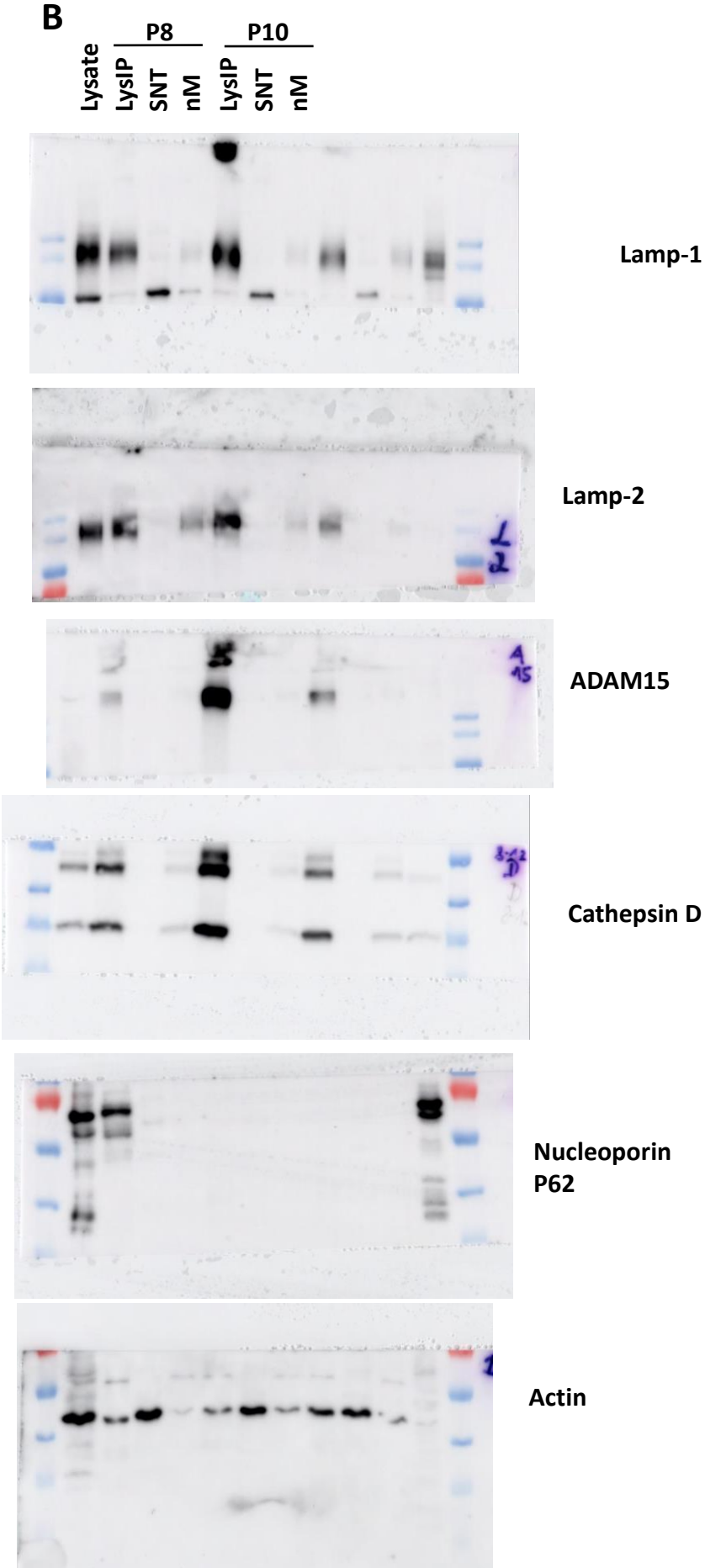

Supplement: Supplementary file 1 — Supplementary Material 1. [file 12964_2025_2530_MOESM1_ESM.pdf]
